# Single-Cell Proteomics Reveals the Defined Heterogeneity of Resident Macrophages in White Adipose Tissue

**DOI:** 10.3389/fimmu.2021.719979

**Published:** 2021-07-26

**Authors:** Inês Félix, Heli Jokela, Joonas Karhula, Noora Kotaja, Eriika Savontaus, Marko Salmi, Pia Rantakari

**Affiliations:** ^1^ Turku Bioscience Centre, University of Turku and Åbo Akademi University, Turku, Finland; ^2^ Research Centre for Infection and Immunity, Institute of Biomedicine, University of Turku, Turku, Finland; ^3^ InFLAMES Research Flagship Center, University of Turku, Turku, Finland; ^4^ Research Centre for Integrative Physiology and Pharmacology, Institute of Biomedicine, University of Turku, Turku, Finland; ^5^ Clinical Pharmacology, Turku University Hospital, Turku, Finland; ^6^ MediCity Research Laboratory, University of Turku, Turku, Finland

**Keywords:** adipose tissue, mass cytometry (CyTOF), developmental origin, obesity, macrophage

## Abstract

Adipose tissue macrophages (ATMs) regulate homeostasis and contribute to the metabolically harmful chronic inflammation in obese individuals. While evident heterogeneity of resident ATMs has been described previously, their phenotype, developmental origin, and functionality remain inconsistent. We analyzed white adipose tissue (WAT) during homeostasis and diet interventions using comprehensive and unbiased single-cell mass cytometry and genetic lineage tracking models. We now provide a uniform definition of individual subsets of resident ATMs. We show that in lean mice, WAT co-harbors eight kinetically evolving CD206^+^ macrophage subpopulations (defined by TIM4, CD163, and MHC II) and two CD206^–^ macrophage subpopulations. TIM4^–^CD163^+^, TIM4^–^CD163^–^ and CD206^–^ macrophage populations are largely bone marrow-derived, while the proliferating TIM4^+^CD163^+^ subpopulation is of embryonic origin. All macrophage subtypes are active in phagocytosis, endocytosis, and antigen processing *in vitro*, whereas TIM4^+^CD163^+^ cells are superior in scavenging *in vivo*. A high-fat diet induces massive infiltration of CD206^–^ macrophages and selective down-regulation of MHC II on TIM4^+^ macrophages. These changes are reversed by dietary intervention. Thus, the developmental origin and environment jointly regulate the functional malleability of resident ATMs.

## Introduction

Obesity and impaired metabolic health are escalating worldwide, and obesity is a significant risk factor for metabolic disorders, including type II diabetes, non-alcoholic fatty liver disease, atherosclerosis, and ischemic cardiovascular disease (1, 2). The tissue most strongly associated with the pathogenesis of obesity is white adipose tissue (WAT). Adipose tissue macrophages (ATMs), the most abundant immune cell type in WAT, have been associated with the development and expansion of adipose tissue, as well as with antigen presentation, iron metabolism, and catecholamine synthesis (3–8). Immunological profiling of obesity has mainly focused on the contribution of the recruited bone marrow-derived macrophages to WAT (9–16), showing that the CCR2-mediated recruitment of monocytes and their differentiation into macrophages (17–19) forms the basis of harmful obesity-associated chronic inflammation of WAT (20, 21). The behavior and identity of the recruited macrophage populations that accumulate in obese adipose tissue are well characterized (15, 18, 19, 22). Diet-induced obesity (DIO) has been suggested to alter ATMs phenotype from an anti-inflammatory (M2) state towards a more pro-inflammatory (M1) activation state that contributes to insulin resistance (15). Inhibition of the accumulation of pro-inflammatory macrophages in WAT results in amelioration of obese conditions and improved metabolic status (18, 23–28). However, further studies are needed to unravel the function and diversity of resident macrophages, especially in steady state lean adipose tissue, to provide knowledge that would shift intervention approaches away from targeting the inflammatory ATMs towards targeting their metabolic reprogramming in order to maintain adipose tissue homeostasis.

Not all tissue-resident macrophages originate from bone marrow-derived monocyte precursors. In numerous tissues, resident macrophages are established during early embryonic development and are self-maintaining locally throughout adult life, with minimal input from circulating monocytes (29–35). Likewise, adipose tissue also contains self-renewing macrophages, which populate adipose tissue independently of the establishment of bone marrow hematopoiesis (10–12, 36, 37). Recently, several studies have addressed the identity of the steady state ATM subpopulations in mice, both at RNA level with single-cell sequencing and protein level with conventional flow cytometry, demonstrating an evident heterogeneity among the resident ATMs in WAT (15, 38–41). However, it is unclear how these different subsets described in various studies using different techniques and markers are related to each other, and detailed analyses of developmental origin and maintenance of resident macrophages in adipose tissue are still lacking. Defining the identity of different resident ATMs in lean WAT is essential to dissect more precisely their role in homeostatic or obesity-related functions.

In the present study, we aimed to unravel the development, identity, and origin of resident ATM populations in lean WAT and their responsiveness to dietary interventions. Using comprehensive and unbiased single-cell analyses in wild type and genetically modified mouse models, we identified the co-existence of multiple distinct resident ATM populations in the lean WAT defined by CD206, TIM4, CD163, and MHC II cell surface markers. The results showed that resident ATMs are a complex pool of embryonic and infiltrating monocyte-derived macrophages. Our analyses reveal for the first time that resident ATM subsets exist both in lean and obese WAT in relatively steady numbers and have unique phenotypic switches compared to the obesity-induced bone marrow-derived macrophages. Our findings provide a unifying approach for using distinct markers to identify resident ATM populations in a lean WAT and suggest a critical role for the origin of macrophages in regulating the function of ATM subpopulations in WAT.

## Materials and Methods

### Mice

The genetic mouse models used in this study were: *Ccr2^−/−^* (stock 004999), *Nur77^−/−^* (stock 006187), *R26R-EYFP* (stock 006148), *Cx3cr1^CreERT2^* (stock 020940), and *Csf1r^CreEsr1^* (stock 019098), purchased from Jackson Laboratories, and *Plvap^tm1Salm^* (*Plvap^−/−^*) mice (42, 43). Wild type (WT) mice, C57BL/6J and C57BL/6N, were acquired from Janvier labs. All mice were kept under 22°C and 12 hours of light and 12 hours dark cycles at the animal facilities of the University of Turku (Turku, Finland). Unless stated otherwise, the animals were fed with standard pellet chow and reverse osmosis water. Only males were used in all experiments, and age-matched WT mice were used as controls in each experiment. Embryonic development was estimated considering the day of a vaginal plug as embryonic day 0.5 (E0.5). Animal experiments were conducted under the revision and approval of the Regional Animal Experiment Board in Finland, according to the 3R-principle and under Animal license numbers 6211/04.10.07/2017 and 14685/2020. All experiments were regulated according to the Finnish Act on Animal Experimentation (497/2013).

### Mass and Flow Cytometry

Both sides of the epididymal white adipose depots (eWAT) were collected to Hank’s Buffered Saline (Sigma-Aldrich, H9394-500ML). The fat depots were minced with scissors and then digested for 1 hour at 37°C, with 1 mg/ml collagenase D (Roche, 1108886601) and 50 µg/ml DNase 1 (Roche, 10104159001). During the digestion, the samples were shaken 2 to 3 times. After homogenization by pipetting, the cell suspension was left for 10 minutes at room temperature (RT) for gravitational separation of the fractions. The stromal vascular fraction (SVF) fraction was filtered through silk (pore size 77 µm), pelleted, washed twice with Hank’s Buffered Saline, and suspended in PBS or FACS buffer.

For mass cytometry analysis, eWAT from both sides of seven 2-week-old and three 5-week-old mice were pooled. For analyses of 8-, 12-, 16- and 23-week-old mice, two donors per data point were used to get a sufficient number of leukocytes. Samples were stained in U-bottom 96-well plates. They were incubated in 2.5 μM Cell-ID Cisplatin (Fluidigm 201064; 5 min, RT) to exclude dead cells and then with anti-CD16/32 (clone 2.4G2, Bio X Cell; 10 min, RT) to block the Fc-receptors. Target antigens were labeled with pools of metal-tagged antibodies listed in **Supplementary Table 1** (RT, 30 min). The samples were finally stained with DNA intercalation solution (1:1000 Cell ID Intercalator-103Rh in MaxPar^®^ Fix and Perm Buffer, cat. 201067; Fluidigm; 1 hour, RT), and fixed by 4% paraformaldehyde solution (PFA; Santa Cruz Biotechnology, cat. sc-281692; overnight, 4°C). Before the acquisition, the cells were washed and resuspended in purified H_2_O.

The samples were run using Fluidigm Helios Mass Cytometer. Data were analyzed using Cytobank software (Cytobank, Inc) or Cytosplore visual analysis system (44, 45). Bead normalized data were gated for single (191Ir^+^) live (Cisplatin^−^) cells, analyzed by viSNE (dimensionality reduction algorithm t-SNE), and clustered in an unsupervised manner using FlowSOM algorithm (Cytobank, https://www.cytobank.org) or HSNE [Hierarchical Stochastic Neighbor Embedding (44)] using Cytosplore.

For fluorescent flow cytometry, the Fc-receptors were blocked the same way as for CyTOF. The staining with fluorochrome-conjugated antibodies was performed at 4°C for 20 min (the antibodies used are listed in **Supplementary Table 1**). Data were acquired with LSR Fortessa flow cytometer (Becton Dickinson) and analyzed using the FlowJo software (FlowJo LLC).

### Lineage Tracking Experiments

To study the embryonic origin of macrophages, *Csf1r^CreEsr1^* male mice were crossed with R26R-EYFP female mice. The plugged females were injected intraperitoneally (i.p.) with tamoxifen (1.5 mg) and progesterone (0.75 mg) at E8.5 to fluorescently label the offspring’s CSF1R^+^ yolk sac (YS) -derived macrophages. Additionally, *Cx3cr1^CreERT2^* male mice were crossed with R26R-EYFP female mice. Pregnant dams were injected i.p. with the same dose of tamoxifen and progesterone as above at E9.5 or E13.5 to induce reporter recombination on the offspring. Both E9.5 and E13.5 tamoxifen pulsings selectively label only YS-derived macrophages since CX3CR1 is not expressed in the fetal liver-derived monocytes or their precursors (31, 46).

### Histologic Analysis

Epididymal fat pad samples were fixed for 48 to 72 hours at RT with 10% Normal Buffered Formalin (Sigma, cat.:HT501128-4L). The specimens were then dehydrated in 50% EtOH to 70% EtOH gradient and kept at 4°C in 70% EtOH until paraffin embedding at Histology core facility of the Institute of Biomedicine, University of Turku, Finland. Four µm sections were cut, dried overnight at 37°C, deparaffinized, rehydrated, stained with Periodic Acid Schiff (Sigma-Aldrich, cat.:395B-1KT), and counterstained with hematoxylin. Slides were mounted with Dibutylphthalate Polystyrene Xylene (DPX; Sigma, cat.:06522-100ML). Stained sections were imaged using Pannoramic 1000 (3DHISTECH) scanner (with a 40x lens) and analyzed using CaseViewer software (3DHISTECH – CaseCenter 2.9 SP1).

### Proliferation Experiments

5- and 16-week-old mice were injected i.p. with 1.2 mg of bromodeoxyuridine (BrdU, BD Pharmingen, cat.: 51-2420KC). Two hours after injection, fat pads were collected and processed as described above for flow cytometry. After standard FACS staining for leukocyte markers, cells were fixed and stained with FITC-conjugated BrdU antibody (BrdU Flow kit, BD Bioscience).

### Diet Experiments

High-fat diet (HFD; Research diets, cat.: D12492, 5.24 kcal/g, 60 kcal% fat) was used to induce obesity, and it was compared with a natural ingredient-based standard diet [Chow; CRM (E), Special Diet Services, cat.: 801730, 3.62 kcal/g, 9.12 kcal% fat] and purified ingredient normal-fat diet (NFD; Research diets, cat.: D12450J, 3.85 kcal/g, 10 kcal% fat). 7 to 8-week-old male mice were weighed and randomly divided into the specific diet groups fed *ad libitum*. After eight weeks of the experimental diets (at the age of 16 weeks), eWAT from Chow and HFD fed group was analyzed to establish a baseline to study the effects of weight loss in obese mice (n = 8). As weight loss models, last 8 weeks of the experiment (from 16 to 23 weeks), a cohort of HFD-fed mice started a metformin treatment incorporated in the diet (HFD + Met, n = 8; Research diets, cat.: D18120701, 60 kcal% fat, average dose 353 mg/kg calculated based on food intake) and another cohort was reversed to Chow (HFD to Chow, n = 10). The remaining three groups [Chow (n = 8), NFD (n = 8), and HFD (n = 16)] continued with their original diets to the end of the experiment.

All diet experiment mice were weighed every two weeks. Glucose tolerance test (GGT) was performed after 14 weeks of diet interventions (22 weeks of age). Before GTT, mice were fasted for four hours, and the basal blood glucose level was measured. Glucose (2.5 g/kg lean mass) was injected intraperitoneally (i.p.), and glucose was measured from a blood sample using a glucometer (Contour XT, Bayer) obtained from the tail vein 20, 40, 60, and 90 minutes after the glucose injection. The fat mass gain was analyzed two times during the diet interventions (8 and 15 weeks from the beginning of diet intervention) by quantitative nuclear magnetic resonance (NMR) scanning (EchoMRI-700, Echo Medical Systems). After euthanasia, eWAT samples were collected, processed, and analyzed as described above.

### Scavenging Experiments

For *in vitro* endocytosis assays, SVF was isolated from eWAT of 5-week-old WT mice (digested as described in “*Flow and Mass Cytometry*”). Negative control cells were kept at +4°C for 1 hour before exposure to the fluorescent cargoes. SVF of one animal was diluted to 200 µl of RPMI-1640 (Sigma, cat.: R5886-500mL) supplemented with 10% FCS and 2 mM L-glutamine (Glutamax, Gibco, cat.: 35050-038), and the cells were seeded to U-bottom 96-well plates. Fluorescein-conjugated dextran (1 mg/mL; 500 kDa; Invitrogen, cat.: D7136) or fluorescent beads (1:1000, 0.002% of solids; FluoSpheres carboxylate, 0.5 µm, yellow-green 505/515; Invitrogen, cat.: F8813) were added to cells. The plates were incubated at +37°C or +4°C for 1 h. After that, the cells were washed with ice-cold PBS to inactivate them metabolically. Flow cytometric staining was performed as described before. To calculate the phagocytosis/endocytosis capacity of TIM4^+^CD163^+^, TIM4^+^, CD163^+^, and TIM4^–^CD163^–^ ATM subsets, the mean intensity fluorescence (MFI) of samples incubated at +37°C was divided by the MFI of samples incubated at +4 °C.

For *in vivo* endocytosis assays, 5- or 22-week-old mice were injected in the lateral tail vein, with 0.8 mg of 500 kDa dextran Fluorescein (Invitrogen, cat.: D7136) or with 0.1 mg Ovalbumin Fluorescein (Invitrogen, cat.: O23020) – 0,02mg chicken egg albumin antibody (Sigma, cat.: C6534-2ML) complex (47). One (for dextran) or two (for the immunocomplex) hours after the injections, the fat pads were collected, processed, and analyzed by flow cytometry, as described above.

### Antigen Processing Experiment

DQ ovalbumin (DQ-OVA, 1mg/ml; Invitrogen, cat.: D12053) is a self-quenched conjugate of ovalbumin that exhibits green fluorescence after proteolytic degradation in the cells. SVF was isolated from eWAT of 5-week-old WT mice and diet experiment mice (Chow, HFD and HFD to Chow) at 30 weeks of age and incubated with the reporter under the conditions described in the “Scavenging experiments”. Preparation for this assay was performed as mentioned for the *in vitro* scavenging tests. DQ-OVA was added into 200 μL of cell suspension at a concentration of 10 μg/mL. Flow cytometric staining was performed as described before. To calculate the proteolytic capacity of ATM subsets, the MFI of samples incubated at +37°C was divided by the MFI of samples incubated at +4°C.

### Statistics

Adult mice were allocated to experimental groups without specific randomization methods. Numeric data are given as mean ± SEM. Comparisons between the genotypes or treatment groups were made using the nonparametric two-tailed Mann-Whitney test, parametric two-tailed t-test, one- or two-way ANOVA test with Bonferroni post-hoc test. GraphPad Prism 9 (GraphPad Software, LLC) was used for the statistical analyses. P-values ≤ 0.05 are considered to be statistically significant.

## Results

### TIM4 and CD163 Define the Resident Macrophage Populations in Adipose Tissue

We applied a single-cell mass cytometry (CyTOF) approach for immune profiling of white adipose tissue (WAT) macrophage populations. Based on previous studies (11, 15, 38, 39, 41, 48), we first designed an extensive antibody panel including the signature markers associated with resident macrophages. We then carried out comprehensive profiling of epididymal white adipose tissue (eWAT) immune cell composition in 2, 5, 8, 12, 16, and 23 weeks old wild type (WT) male mice on a standard diet (referred hereafter as Chow; **Supplementary Figure 1A**). To visualize the overall immune cell profile of eWAT, we applied the t-Distributed Stochastic Neighbor Embedding (tSNE; 49) dimensionality reduction approach to all CD45^+^ cells (**Supplementary Figures 1B, C**). Across all samples, CD11b^+^ myeloid cells, including macrophages (CD64^+^F4/80^+^), monocytes (Ly6C^+^), dendritic cells (CD11c^+^), granulocytes (Ly6G^+^), and eosinophils (SiglecF^+^), were identified as the dominant leukocyte population (together representing 53 to 60% of all CD45^+^ cells). In addition, T- (CD4^+^ and CD8^+^) and B-lymphocytes (B220^+^), CD11b^−^ dendritic cells (CD11b^−^CD11c^+^), and unclassified CD45^+^ cells were detectable in eWAT at all time points studied (**Supplementary Figure 1C**).

**Figure 1:**
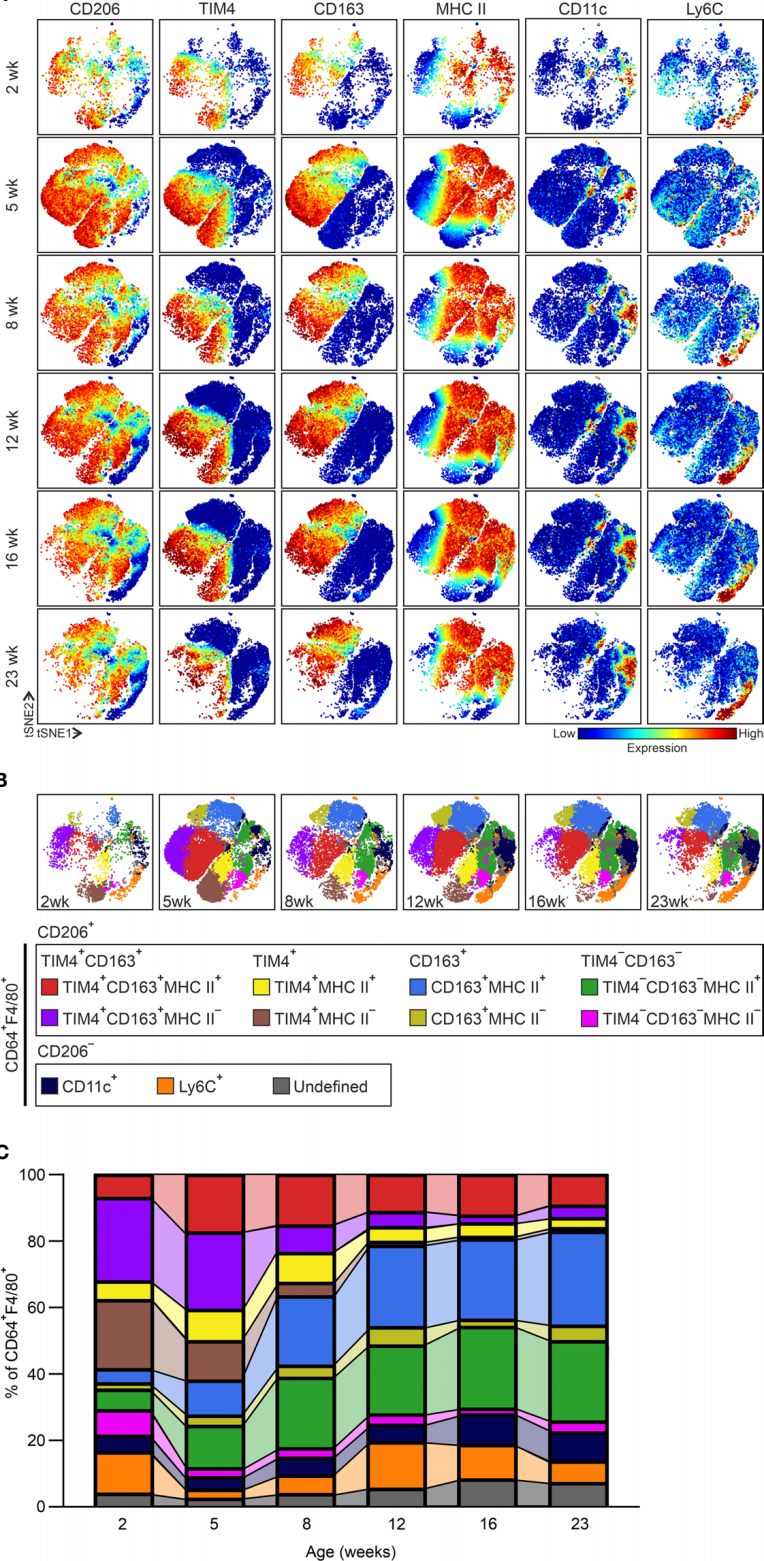
Adipose tissue macrophages (ATMs) evolve during postnatal development. **(A)** Representative viSNE plots of CD45^+^CD64^+^F4/80^+^ macrophages of epididymal white adipose tissue (eWAT) in 2-, 5-, 8-, 12-, 16- and 23-week-old wild-type (WT) mice. The color code indicates the expression level of a given marker from low (blue) to high (red). **(B)** Representative unsupervised FlowSOM analysis of eWAT CD45^+^CD64^+^F4/80^+^ macrophage cell clusters at the indicated time points. Each FlowSOM metacluster (subpopulations) is indicated in different colors. **(C)** Frequencies of the macrophage subpopulations in WT eWAT based on the FlowSOM analyses at the indicated time points. Each FlowSOM metacluster (subpopulation) is represented by an individual color depicted in the columns (from **B**). Data are from n = 14 (2 wk), 12 (5 wk), n = 6 (8, 12, and 16) or n = 8 (23 wk) mice.

We then focused on the immunophenotypic characteristics and the kinetics of eWAT tissue-resident macrophages in more detail from juvenile (2- to 5-week-old) to adult (8- to 23-week-old) WT mice. We manually gated the macrophages from CD45^+^ cells based on the commonly used canonical macrophage markers, CD64^+^ and F4/80^+^ (**Supplementary Figure 1B**). As expected, most steady state eWAT macrophages from 2 to 23 weeks expressed CD206, a well-characterized M2-like macrophage marker, and only a minor fraction of CD64^+^F4/80^+^ macrophages were CD206 negative in a lean eWAT (**Figure 1A**). To avoid bias through manual gating, we performed unsupervised clustering of CD64^+^F4/80^+^ cells with the FlowSOM algorithm (50). This self-organizing map on top of tSNE visualization distinguished several macrophage subclusters, each expressing unique surface marker profiles (**Figures 1A, B** and **Supplementary Figure 2**). Among the CD206^+^CD64^+^F4/80^+^ tissue-resident macrophages, four main clusters of cells were observed based on their differential expression of TIM4 and CD163. For brevity, the TIM4^+^CD163^+^, TIM4^+^CD163^−^, TIM4^−^CD163^+^ and TIM4^−^CD163^−^ populations will be called hereafter TIM4^+^CD163^+^, TIM4^+^, CD163^+^, and TIM4^−^CD163^−^, respectively. Each of these four major populations was further subdivided into two clusters based on major histocompatibility complex II (MHC II) expression (**Figure 1B** and **Supplementary Figure 2**). All MHC II^+^ clusters also expressed higher levels of Siglec1 (CD169) than MHC II^−^ sub-clusters (**Supplementary Figure 2**). Interestingly, CX3CR1 expression was detected mainly in TIM4^−^CD163^−^MHC II^+^ cluster and in a small proportion of cells in the CD163^+^MHC II^+^ cluster (**Supplementary Figure 2**).

Kinetic analyses showed that several macrophage subpopulations underwent characteristic alterations in frequency between the different time points (**Figure 1C** and **Supplementary Figures 3A–F**). In both TIM4^+^CD163^+^ and TIM4^+^ ATM populations, the frequency of MHC II positive macrophages increased from 2 to 5 weeks of age and thereafter started to decrease slowly. In contrast, the MHC II negative TIM4^+^CD163^+^ and TIM4^+^ ATMs, the major subpopulations at juvenile (2- to 5-week-old) time points, strongly diminished throughout the postnatal development (**Figure 1C** and **Supplementary Figures 3A, B**). Among CD163^+^ and TIM4^−^CD163^−^ macrophages, on the other hand, the frequency of MHC II positive cells grew significantly by age (**Figure 1C** and **Supplementary Figures 3C, D**). While the majority of CD64^+^F4/80^+^ cells were CD206 positive, a minor percentage of CD206 negative cells were also identified (**Figures 1A–C**). Consistent with other reports (15, 39), the CD206^−^CD64^+^F4/80^+^ cells in lean mice included a small CD11c and Ly6C^+^ positive ATM clusters (**Figures 1A–C** and **Supplementary Figure 2**), likely representing monocyte-derived cells. The frequency of CD11c^+^ ATMs remained relatively steady, while the frequency of Ly6C^+^ ATMs fluctuated throughout the postnatal development (**Figure 1C** and **Supplementary Figures 3E, F**). Notably, although most CD11c^+^ cells were observed to be CD206 negative, low numbers of TIM4^+^CD163^+^ and CD163^+^ cells also expressed CD11c (**Supplementary Figure 2**).

Previous work has reported that most of the resident ATMs in lean mice are associated with adipose tissue vasculature. The vasculature-associated macrophages (VAMs) were identified with the expression of CD206, TIM4, and MHC II surface markers (11). To compare our data with the identified VAM macrophage populations, we manually gated our mass cytometry data with the same gating strategy used to determine the VAMs (**Supplementary Figure 4A**). We observed that all the populations recognized previously, VAM1, VAM2, pre-VAM and double positive (DP) macrophages, actually included cells from all main CD206^+^ (TIM4^+^CD163^+^, TIM4^+^, CD163^+^, and TIM4^−^CD163^−^) clusters identified with our unsupervised computational approach (**Supplementary Figure 4B**). In many tissues, LYVE1 positive macrophages are assigned to be the vessel-associated macrophages (51), and scRNA-seq experiments have correspondingly shown that the ATMs in adult lean mice express *Lyve1* (11, 38). Therefore, we next studied LYVE1 from our data. We found that LYVE1^+^ macrophages did not represent a separate cluster but were identified in small subpopulations within each of the four major CD206^+^ ATM populations at all time points studied (**Supplementary Figures 4C, D**). Likewise, the LYVE1 positivity was found among all VAM1, VAM2, pre-VAM, and DP macrophage populations (**Supplementary Figure 4B**). Together these single-cell mass cytometric analyses show that the division of ATM subclasses in lean WAT is much more complex than previously appreciated and pointed out the need for higher dimensional flow cytometry and computational methods to capture the full heterogeneity of ATMs. Our mass cytometric analyses provide the first high-resolution map of the development of ATMs in eWAT and identify the optimal surface markers for identifying the distinct macrophage subtypes.

### Bone Marrow-Derived CCR2 Dependent Monocytes Account for TIM4 Negative Tissue-Resident Macrophages in White Adipose Tissue

**Figure 2:**
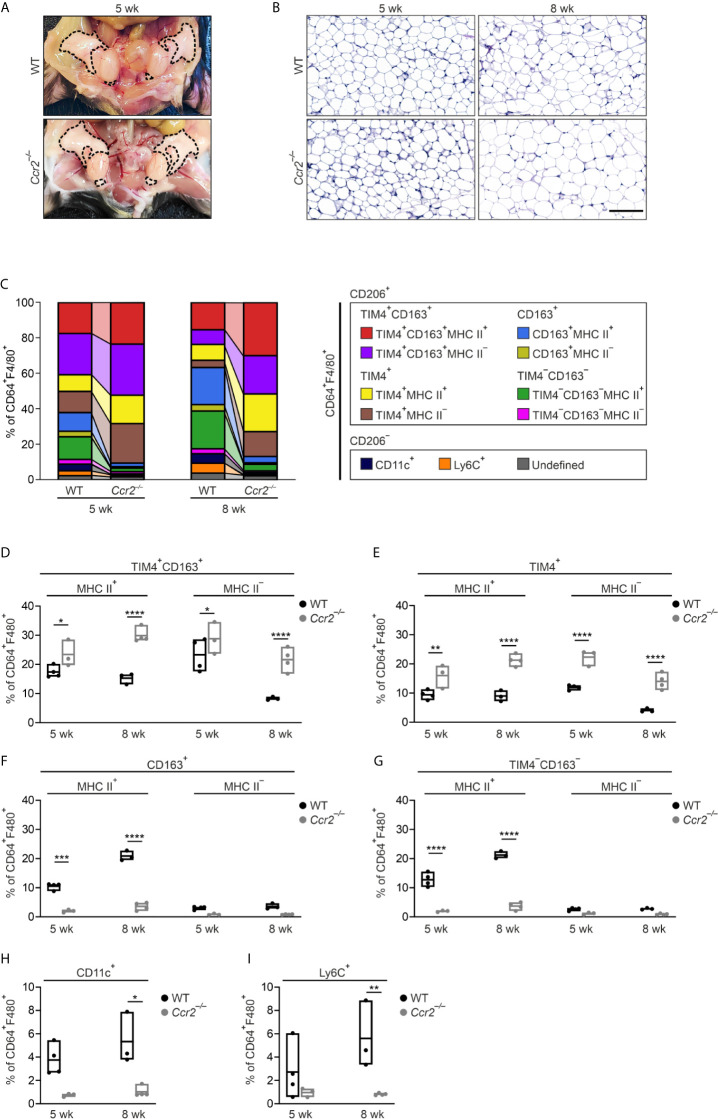
CCR2-dependent trafficking of bone marrow-derived monocytes contributes differentially to eWAT macrophage subpopulations. **(A)** Representative macroscopic images of eWAT in 5-week-old wild type (WT) and *Ccr2* ^-/-^ mice. Epididymal fat pads are outlined with a dashed line. **(B)** Representative PAS-stained histology of eWAT in 5- and 8-week-old WT and *Ccr2* ^-/-^ mice. Scale bar 100 µm. **(C)** Frequencies of macrophage subpopulations in eWAT of 5- and 8-week-old WT and *Ccr2* ^-/-^ mice based on the FlowSOM analyses at the indicated time points. Each FlowSOM metacluster (subpopulation) is represented by an individual color depicted in the columns. **(D–I)** Frequencies of eWAT macrophage subpopulations in *Ccr2* ^-/-^ and WT mice. The quantitative data are shown as mean ± SEM (*P ≤ 0.0332, **P ≤ 0.0021, ***P ≤ 0.0002, ****P ≤ 0.0001, two-way ANOVA with Bonferroni post-hoc test). Each data point represents a pooled eWAT from 3 mice for 5 wk and 2 mice for 8 wk. All mass cytometry data are from 2 (8 wk WT) or 3 (5 wk WT, and 5 and 8 wk *Ccr2* ^-/-^) independent experiments.

The circulating bone marrow-derived monocytes are known to heavily replenish macrophage pools in WAT in obesity (19). To address the contribution of bone marrow-derived monocytes to eWAT tissue-resident macrophage subpopulations in steady state, we used *Ccr2*- and *Nur77*-deficient mice manifesting with low numbers in circulating classical (Ly6C^High^) and patrolling (Ly6C^Low^) monocytes, respectively (52, 53). The total body weights and eWAT morphology of WT and *Ccr2^−/−^* male mice were comparable (**Figures 2A, B** and **Supplementary Figure 5A**), except for lighter eWAT fat pads in over 12-week-old *Ccr2^−/−^* mice (**Supplementary Figure 5B**), which is a known phenotype of *Ccr2* deficient mice (18). FlowSOM clustering analysis of 5- and 8-week-old *Ccr2^−/−^* mice eWAT showed that the frequency of TIM4^+^CD163^+^ and TIM4^+^ ATMs (MHC II positive and negative subpopulations) was increased (**Figures 2C–E**; and **Supplementary Figure 5C**). On the other hand, *Ccr2* deletion resulted in a striking loss of CD163^+^MHC II^+^ and TIM4^−^CD163^−^MHC II^+^ ATM populations that were not recovered upon aging (**Figures 2C, F, G**). Additionally, CD206^−^CD11c^+^ and CD206^−^Ly6C^+^ macrophage populations were significantly diminished in *Ccr2* deficient mice compared to WT controls (**Figures 2H, I**). On the contrary, the contribution of bone marrow-derived patrolling Ly6C^Low^ monocytes in the generation of ATMs was not substantial. A slight decrease in the frequency of CD163^+^MHC II^+^ and TIM4^−^CD163^−^MHC II^+^ ATMs was seen only at 8-week time point in *Nur77*-deficient mice. Interestingly, the Ly6C^+^ cell populations increased in *Nur77^−/−^* mice (**Supplementary Figures 5C–J**). These genetic models collectively show that the bone marrow-derived CCR2 dependent Ly6C^High^ monocytes contributed greatly not only to the CD206^−^CD11c^+^ and CD206^−^Ly6C^+^ ATM populations but also control the generation of the two TIM4 negative ATM populations (CD163^+^MHC II^+^ and TIM4^−^CD163^−^MHC II^+^) in lean mice.

### Adipose Tissue TIM4^+^CD163^+^ Macrophages Are Embryonically Derived

**Figure 3:**
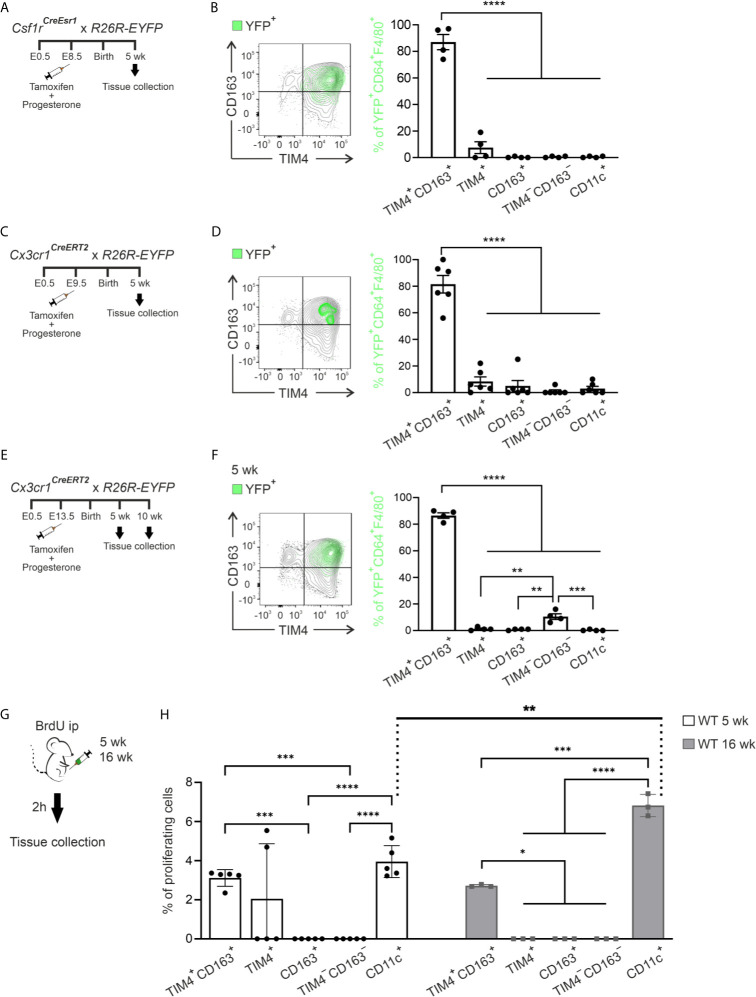
Yolk sac-derived macrophages give rise to self-renewing tissue-resident ATMs. **(A, B)** The experimental outline **(A)** and representative FACS plot and kinetic quantifications **(B)** of yolk sac‐derived macrophages in ATMs of *Csf1r^CreEsr1^; R26R-EYFP* mice. **(C, D)** The experimental outline **(C)** and representative FACS plot and quantifications **(D)** of yolk sac‐derived macrophages in ATMs with the early conversion of *Cx3cr1^CreERT2^; R26R-EYFP* reporter mice. **(E, F)** The experimental outline **(E)** and representative FACS plot and quantifications **(F)** of yolk sac‐derived macrophages in ATMs of the *Cx3cr1^CreERT2^; R26R-EYFP* mice with the late conversion of *Cx3cr1^CreERT2^; R26R-EYFP* reporter mice. **(B, D, F)** The plots show the backgating of the YFP^+^ cells (green) on the different ATM populations. The quantifications show the frequency of CD45^+^YFP^+^CD64^+^F4/80^+^ cells in each macrophage population. **(G)** The experimental outline shows i.p. administration of BrdU to 5- and 16-week-old wild type (WT) mice and harvesting of ATMs after 2 hours. **(H)** Frequencies of BrdU positive cells in ATM populations. The quantitative data are shown as mean ± SEM [*P ≤ 0.0332, **P ≤ 0.0021, ***P ≤ 0.0002, ****P ≤ 0.0001, one-way ANOVA **(B, D, F)** and two-way ANOVA **(H)** with Bonferroni post-hoc test]. Each data point represents one mouse. All flow cytometry data are from 2 (**B, D, F**, and **H** for 5 wk) or 1 (**H** for 16 wk) independent experiments.

The embryonic-derived macrophage progenitors, either yolk sac or fetal liver-derived, contribute to tissue-resident macrophage populations in multiple organs (46, 54–59). Therefore, we carried out the cell-fate mapping experiments to address the contribution of the yolk sac-derived precursor to the ATM populations in lean eWAT. To identify yolk sac-derived macrophages (60), we induced YFP conversion in *Csf1r^CreEsr1^;R26R-EYFP* -reporter mice by a single tamoxifen injection at E8.5 (**Figure 3A**) and used flow cytometry for the disentanglement of YFP positive cells in adult mice (**Supplementary Figure 6A**). We found YFP^+^ cells solely in TIM4^+^CD163^+^ positive macrophage population in 5-week-old mice (over 80% of YFP^+^CD64^+^F4/80^+^ cells were TIM4^+^CD163^+^ macrophages). From the rest of the ATMs, only the TIM4^+^ population had a small percentage of YFP^+^ cells, while CD163^+^, TIM4^−^CD163^−^, and CD11c^+^ ATMs were completely YFP negative (**Figure 3B**). As an alternative model to label yolk sac-derived macrophages, we pulsed *Cx3cr1^CreERT2^;R26R-EYFP* -reporter mice (31, 46, 61) with tamoxifen at E9.5 (**Figure 3C**). The results confirmed the embryonic origin of TIM4^+^CD163^+^ cells and the lack of contribution of yolk sac-derived cells to the other macrophage populations (**Figure 3D**). When cells were converted later (at E13.5) in *Cx3cr1^CreERT2^;R26R-EYFP* (31, 46, 61) mice (**Figure 3E**), the vast majority of YFP^+^ cells were still found in TIM4^+^CD163^+^ subpopulation, both in 5- and 10-week-old mice (**Figure 3F** and **Supplementary Figure 6B**). Interestingly, the conversion at E13.5 resulted in a slight YFP positivity among TIM4^−^CD163^−^ ATMs in 5-week-old mice (**Figure 3F**). We noticed that the absolute cell number of YFP^+^ cells dramatically decreased in the 10-week-old mice (**Supplementary Figure 6C**), indicating restricted survival of yolk sac-derived macrophages in aging mice.

To gain additional insight into the development of ATMs, we analyzed *Plvap^−/−^* mice, which shows a selective reduction of fetal liver-derived macrophages in many tissues (42, 43). At the age of 5 weeks, *Plvap^−/−^* mice had significantly lower body weight but comparable relative weight and morphology of eWAT to WT mice (**Supplementary Figures 6D–F**). We found an overall reduction of CD64^+^F4/80^+^ macrophages in *Plvap* ^-/-^ mice eWAT (**Supplementary Figure 6G**). More detailed analysis revealed that the frequency of the TIM4^+^CD163^+^ positive macrophage population was reduced in *Plvap* deficient mice compared to the WT littermates (**Supplementary Figures 6H, I**). Together with the lineage tracking experiments, these results indicate that embryonic macrophage precursors, both from the yolk sac and fetal liver origin, enter the eWAT during embryonic development and persist in eWAT after birth. Collectively, the data support the embryonic origin of the TIM4^+^CD163^+^ ATMs.

The dynamic changes in ATM subpopulations during aging might be due to local proliferation. To assess this possibility, we quantified the proportion of proliferating ATM cells *in vivo* with 5-bromo-2`-deoxyuridine (BrdU) labeling (**Figure 3G**). TIM4^+^CD163^+^ and CD11c^+^ ATM subpopulations consistently had BrdU^+^ cells at the age of 5 and 16 weeks. At the age of 16 weeks, resident CD11c^+^ cells proliferated faster than any other ATMs. In contrast, we did not observe any reproducible macrophage proliferation in TIM4^+^, CD163^+^, or TIM4^−^CD163^−^ ATM populations (**Figure 3H**). The data collectively reveal that TIM4^+^CD163^+^ and CD11c^+^ macrophages in lean eWAT undergo *in situ* proliferation to retain the population density at a steady state. Conversely, the expansion of TIM4^−^CD163^−^ and CD163^+^ populations depends mainly on CCR2-dependent recruitment of bone marrow-derived circulating blood monocytes rather than local proliferation.

### The Major Tissue-Resident Macrophage Subpopulations Persist in Adipose Tissue During Metabolic Alterations

**Figure 4:**
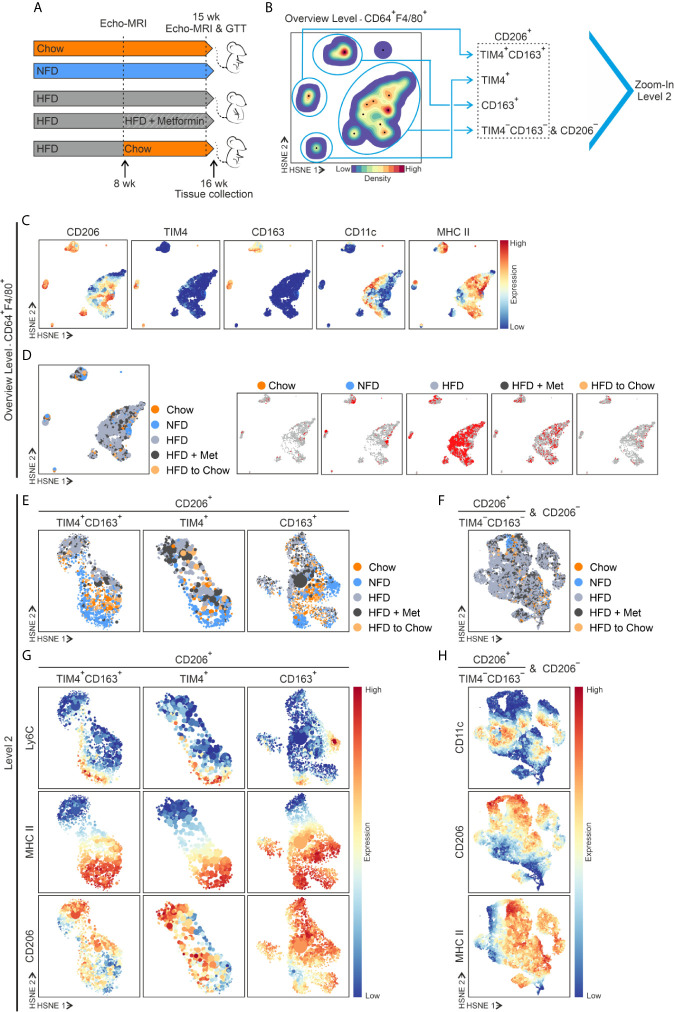
Diet interventions induce alterations in ATMs. **(A)** Experimental setup of diet interventions. The standard diet is referred to as Chow, normal-fat diet as NFD, and high-fat diet as HFD. In the weight loss models, HFD fed mice were administrated with metformin (referred to as HFD + Met), or the diet was switched to Chow (referred as HFD to Chow) after 8 weeks on HFD (at 16 weeks of age). **(B)** Overview HSNE level (Level 1) embedding of CD64^+^F4/80^+^ ATMs in eWAT at the end of the dietary experiments (cells from all study groups superimposed). HSNE level embedding shows density features (blue, low density; red, high density) depicting the local probability density of cells. The black dots indicate the centroids of identified cluster partitions. Blue encirclement indicates manual selection of the major ATM populations representing TIM4^+^CD163^+^, TIM4^+^, CD163^+^, and the merged TIM4^−^CD163^−^ and CD206^−^ ATMs. **(C)** Expression of the indicated markers. **(D)** The left-hand blot: Contribution of cells from the five different diet groups (color-coded as indicated) to the different HSNE density clusters (dark orange for Chow, light orange for HFD to Chow, light blue for NFD, light gray for HFD, and dark gray for HFD + Met). In the five other plots: Each diet is embedded individually, and the red color presents the cells in the indicated diet group, and the gray color shows cells in the whole data set. **(E)** HSNE embedding representing a selection of TIM4^+^CD163^+^, TIM4^+^, and CD163^+^ ATMs (Zoom level 2), demonstrating different diet groups with different colors as indicated. **(F)** Selection of merged CD206^+^TIM4^−^CD163^−^ and CD206^−^ ATMs in HSNE embedding representing different diet groups with different colors as indicated. **(G)** Expression of the indicated markers in TIM4^+^CD163^+^, TIM4^+^, and CD163^+^ ATMs. **(H)** Expression of the indicated markers in merged CD206^+^TIM4^−^CD163^−^ and CD206^−^ ATMs. Indicated markers’ expression intensity is shown from low (blue) to high (red). All mass cytometry data are from n = 8 (Chow), n = 8 (NFD), n = 16 (HFD), n = 8 (HFD + Met), or n= 10 (HFD to Chow) mice.

To study the influence of different diet interventions on eWAT resident ATM populations, we set up several models. We fed WT male mice for 16 weeks (from 8 weeks to 23 weeks of age) with an obesogenic high-fat diet (HFD) and compared them with mice fed with natural ingredient-based chow diet (Chow) and purified ingredient normal-fat diet resembling chow in the levels of macronutrients, but HFD in the sources of other nutrients (NFD; **Figure 4A** and **Supplementary Figures 7A–C**). Moreover, we established two different weight loss models to explore further how weight loss will impact the ATM subpopulations. To this end, mice were first fed with HFD to induce obesity (8-week diet) and, after that, mice were assigned either to metformin treatment (HFD + Met), known to reduce body weight and improve insulin sensitivity (62), or switched to standard chow (HFD to Chow; **Figure 4A** and **Supplementary Figures 7A–C**).

To identify ATM populations under different diets and to avoid the possible effect of downsampling, we first implemented the Hierarchical Stochastic Neighboring Embedding (HSNE) for the mass cytometry data (44, 63). ATM cells from the diet experiments were analyzed with the Cytosplore single-cell analysis framework. At the overview level, the CD64^+^F4/80^+^ ATMs analysis verified that the same major CD206 positive macrophage populations TIM4^+^CD163^+^, TIM4^+^, and CD163^+^ identified in steady state, also existed in all diet groups (**Figures 4B, C**). However, in HFD and HFD + Met, the TIM4^−^CD163^−^ population was merged with a considerably increased inflammatory CD11c cell population (**Figures 4B–D**).

We then selected and explored all subpopulations separately by generating new higher resolution embeddings (**Figures 4E, F**). The result showed that the frequency of CD163^+^ macrophages decreased in HFD and HFD + Met groups and TIM4^+^CD163^+^ cells showed a similar tendency compared to Chow (**Supplementary Figure 8A**). However, this was caused by the influx of inflammatory CD11c^+^ cells as, despite the large individual variability, the cell number stayed relatively steady in TIM4^+^CD163^+^, TIM4^+^, and CD163^+^ populations, regardless of the diet intervention (**Supplementary Figure 8B**). The absolute cell number data suggested that tissue-resident ATMs do not respond to HFD with increased proliferation or apoptosis. Interestingly, only the NFD diet showed a trend towards increased cell counts in TIM4^+^CD163^+^ and CD163^+^ populations (**Supplementary Figure 8B**).

Overlayed visualization of different diet groups showed several diet-specific regions in TIM4^+^CD163^+^, TIM4^+^, and CD163^+^ATM subpopulations (**Figures 4E, G** and **Supplementary Figure 8C**). For instance, the NFD ATMs clustered separately because of their high Ly6C expression (**Figures 4E, G**), indicating that monocyte differentiation to macrophages occurs during the NFD diet. In the Chow, NFD, and HFD to Chow groups, we found that the TIM4^+^CD163^+^, TIM4^+^ and CD163^+^ ATMs had high MHC II expression. In contrast, the HFD and HFD + Met treatment induced a phenotypic switch in ATMs, resulting in a downregulation of MHC II expression (**Figures 4E, G**). Furthermore, in the HFD, HFD + Met, and NFD groups, the TIM4^+^CD163^+^ MHC II^−^ and CD163^+^MHC II^−^ macrophage populations had higher expression of CD206 (**Figures 4E, G**), which is commonly considered as an M2-like macrophage marker (64). The TIM4^−^CD163^−^ together with CD206^−^ ATM populations underwent remarkable remodeling during the diet alterations (**Figures 4F, H**). Among these cells, HFD induced a vast expansion of inflammatory CD11c^+^ ATM cells, which is in line with earlier published literature (22, 65, 66). However, a similar effect was also seen in the HFD + Met group (**Figures 4F, H**).

Interestingly, the expression of CD115 and CX3CR1 was induced within the merged TIM4^−^CD163^−^ and CD206^−^ ATMs in HDF and HFD + Met groups (**Supplementary Figure 8D**). In contrast, the switch from HFD to Chow decreased the number of TIM4^−^CD163^−^ and CD206^−^ ATM cells back to the normal state (**Supplementary Figure 8B**). Together these results indicate that while the HFD induces a major influx of macrophages, the resident ATMs persist in the eWAT despite the nutritional challenges or dietary or pharmacological anti-obesity treatments.

### Obesity Induces TIM4^+^CD163^+^ and TIM4^+^ Macrophages to Undergo MHC II Reprogramming

**Figure 5:**
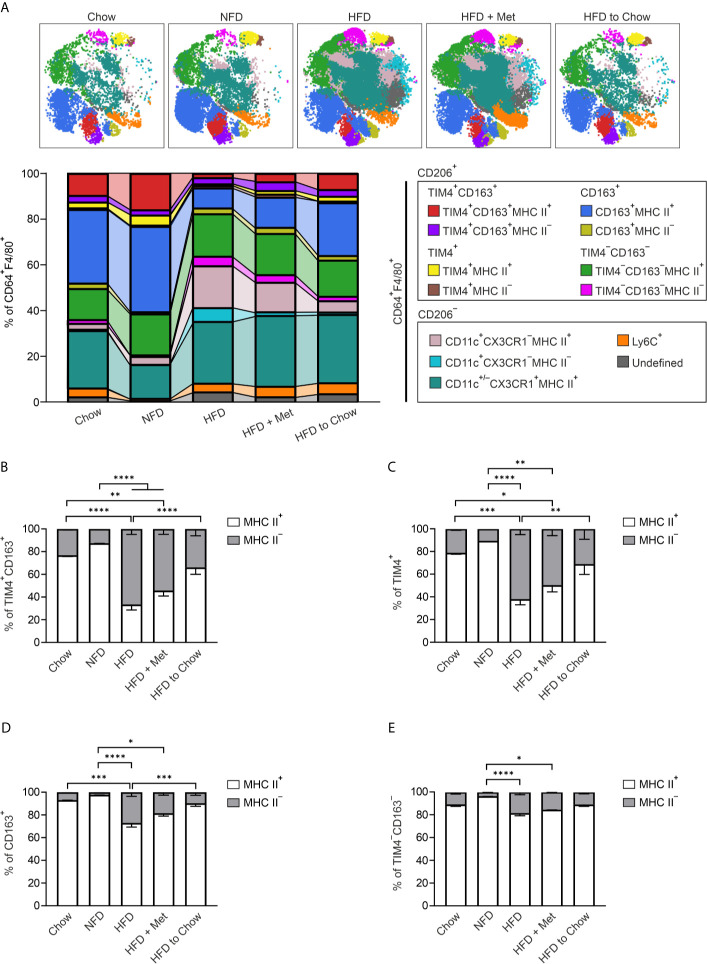
Obesity induces reversible down-regulation of MHC II in resident ATMs. **(A)** Representative FlowSOM maps and frequencies of CD45^+^CD64^+^F4/80^+^ macrophage cell clusters in eWAT of wild type (WT) mice with different dietary interventions; standard diet (Chow), normal-fat diet (NFD), high-fat diet (HFD), or weight loss models; metformin-treated mice on HFD (HFD + Met) or HFD fed mice switched to standard diet after 8 weeks (HFD to Chow). Individual FlowSOM metaclusters (subpopulations) are indicated in different colors. **(B–E)** Frequencies of MHC II^+^ and MHC II^−^ cells in different ATM subpopulations of mice on different dietary interventions. The quantitative data are shown as mean ± SEM (*P ≤ 0.0332, **P ≤ 0.0021, ***P ≤ 0.0002, ****P ≤ 0.0001, two-way ANOVA with Bonferroni post-hoc test). All mass cytometry data are from n = 8 (Chow), n = 8 (NFD), n = 16 (HFD), n = 8 (HFD + Met), or n = 10 (HFD to Chow) mice and from 2 independent experiments.

For quantifying the MHC II expression, we applied viSNE followed by an unsupervised FlowSOM algorithm to cluster the CD64^+^F4/80^+^ ATMs from the different diet interventions. FlowSOM identified 13 metaclusters, of which main TIM4^+^CD163^+^, TIM4^+^, CD163^+^, TIM4^−^CD163^−^ and CD11c^+^ ATM populations were identified (**Figure 5A** and **Supplementary Figure 9A**). Quantitative analyses revealed the expected increase in the frequency of CD11c positive cell populations, while Ly6C cells stayed steady (**Supplementary Figures 9B, C**). More interestingly, quantitative analyses showed that all ATM subpopulations were mainly MHC II positive in Chow and NFD diets (**Figures 5B–E**). In contrast, the HFD induced a robust phenotype switch in TIM4^+^CD163^+^ and TIM4^+^ macrophages with significant downregulation of MHC II expression (**Figures 5B, C**). This phenotypic change was already detectable after an 8-week HDF diet (**Supplementary Figure 9D**). This switch appeared to be specific as the alterations in the expression of MHC II in the bone marrow-derived CD163^+^ and TIM4^−^CD163^−^ macrophages, although significant, were substantially smaller (**Figures 5D, E**). Interestingly, we observed that the diet switch from HFD to Chow returned the MHC II expression of TIM4^+^CD163^+^ and TIM4^+^ macrophages to steady state, while the metformin treatment did not (**Figures 5B, C**). This macrophage phenotype change was associated with a significant decrease in adipocyte size (**Supplementary Figure 9E**). Together these results suggest that resident ATM populations have an origin-dependent MHC II response to diet-induced obesity.

### All WAT Macrophage Subtypes Are Phagocytic and Endocytic Cells With the Capacity to Process Antigens

**Figure 6:**
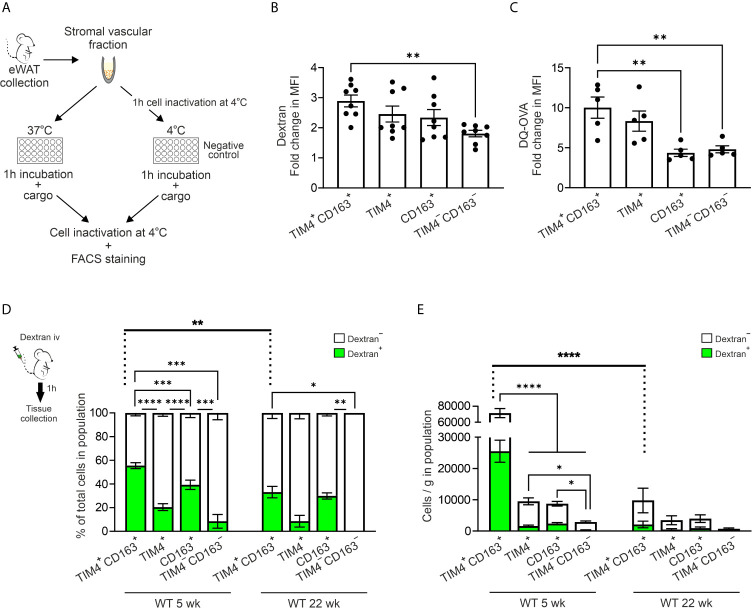
TIM4^+^CD163^+^ and TIM4^+^ ATMs are efficient in scavenging and have an antigen processing capacity. **(A)** The experimental setup for measuring the *in vitro* engulfment of 0.5 µm fluorescent beads, 500 kDa fluorescent dextran, or DQ ovalbumin by different macrophage subtypes isolated from eWAT of 5 or 30-week-old wild type (WT) mice. **(B)** Endocytosis of dextran by the different macrophage subpopulations [fold change in mean fluorescence intensity (MFI) at 37°C versus background at 4°C]. **(C)** Antigen processing capacity of the different macrophage subpopulations (fold change in MFI of DQ-OVA at 37°C versus background at 4°C). **(D)** Scavenging of dextran *in vivo*. The experimental setup for studying scavenging (intravenously administered fluorescent 500 kDa dextran). The quantifications show the frequencies of dextran positive and dextran negative cells within TIM4^+^CD163^+^, TIM4^+^, CD163^+^, and TIM4^−^CD163^−^ ATM populations of 5- and 22-week-old WT mice. **(E)** Analyses of the absolute numbers of dextran positive and dextran negative cells from **(D)** The quantitative data are shown as mean ± SEM (*P ≤ 0.0332, **P ≤ 0.0021, ***P ≤ 0.0002, ****P ≤ 0.0001, one- **(B, C)** or two-way **(D, E)** ANOVA with Bonferroni post-hoc test). Each data point represents one mouse in **(B, C)** Dextran *in vivo* experiment **(D, E)** data are from n = 9 (5 wk) or n = 4 (22 wk) mice. All flow cytometry data are from 1 (**D** for 22 wk, and **E** for 22 wk) or 2 (**B–E** for 5 wk) independent experiments.

To study the phagocytic and endocytic ability of ATMs *in vitro*, we incubated the isolated cells with fluorescent beads (0.5µm) or fluorescent dextran (500 kDa; **Figure 6A** and **Supplementary Figure 10A**). The results revealed that all four main ATM subpopulations in the eWAT were able to phagocytose the fluorescent beads (**Supplementary Figure 10B**). Furthermore, when the cells were incubated with dextran *in vitro*, endocytosis took place in all ATM subpopulations, although the TIM4^+^CD163^+^ cells had higher endocytic capacity than the TIM4^−^CD163^−^ cells (**Figure 6B** and **Supplementary Figure 10C**). Within TIM4^+^, CD163^+^, and TIM4^−^CD163^−^ subpopulations, the MHC II positive cells showed higher efficacy in endocytosis of dextran, while in TIM4^+^CD163^+^ ATMs, both MHC II^+^ and MHC II^−^ cells showed equal endocytic capacity (**Supplementary Figure 10D**). Altogether, these *in vitro* results support that all populations we have assigned as macrophages in eWAT are indeed genuine macrophages with phagocytic and endocytic capacity.

To study the ability of isolated resident ATMs to process soluble antigens, we used DQ-ovalbumin conjugated with BODIPY FL dye (DQ-OVA) as a surrogate antigen. This self-quenched reporter emits fluorescence upon proteolytic degradation (67). Interestingly, all ATM subsets appeared to have a capacity to endocytose and process antigens when studied *in vitro* after one-hour incubation with DQ-OVA (**Figure 6C** and **Supplementary Figure 10E**). However, at the age of 5 weeks, especially TIM4^+^CD163^+^ ATMs degraded DQ-OVA significantly higher degree than CD163^+^ or TIM4^−^CD163^−^ cells (**Figure 6C**). The MHC II^+^ cells displayed more predominant proteolytic capacity in the TIM4^+^ resident ATM population, while in all the other resident ATMs, both MHC II^+^ and MHC II^−^ cells showed equal antigen processing capability (**Supplementary Figure 10F**). We similarly performed *in vitro* DQ-OVA experiment with ATMs isolated from different diet experiments. The result revealed that contrary to earlier timepoint, CD163^+^ and TIM4^−^CD163^−^ ATMs showed the most active antigen processing in the aged animals (Chow group; **Supplementary Figure 10G**). However, no difference in antigen processing between resident ATMs in the different diet intervention groups was observed (**Supplementary Figures 10G–I**). Thus, all ATM subpopulations are capable of antigen processing. While aging impacts the antigen processing capability of resident ATMs, it is not chanced due to metabolic challenges.

### Embryonic-Derived TIM4^+^CD163^+^ Macrophages Are Superior in Scavenging *In Vivo*

Unlike *in vitro*, macrophages’ scavenging properties *in vivo* are also regulated by the proximity of macrophage subsets to the antigen’s entrance routes into adipose tissue. Therefore, to evaluate possible functional differences of macrophage subpopulations *in vivo*, we intravenously (i.v.) administered fluorescently labeled 500 kDa dextran or fluorescent immune complex (OVA-SIC) to 5-week-old WT mice (**Figure 6D** and **Supplementary Figure 10J**). Interestingly, TIM4^+^CD163^+^ and CD163^+^ populations were the most effective ATMs to scavenge dextran from the blood (**Figure 6D**). When the total cell numbers were analyzed, only the embryonic-derived TIM4^+^CD163^+^ ATM population showed noteworthy scavenging of dextran *in vivo* at 5 weeks of age (**Figure 6E**). Notably, the ability of TIM4^+^CD163^+^ ATMs to bind/uptake dextran was significantly reduced at 22 weeks of age, indicating an age-dependent adjustment in the functionality of embryonic-derived ATMs (**Figures 6D, E**). When analyzing *in vivo* OVA-SIC, the binding/up-take was readily detected in F4/80^+^ macrophages in the spleen, but there was only minimal OVA-SIC accumulation in eWAT macrophages (**Supplementary Figure 10J**). Nevertheless, the only OVA-SIC binding cells visible in eWAT were TIM4^+^CD163^+^ macrophages (**Supplementary Figure 10J**). These *in vivo* findings demonstrate a superior scavenging capacity of embryonic-derived TIM4^+^CD163^+^ tissue-resident macrophages *in vivo* in steady state conditions.

## Discussion

Although resident adipose tissue macrophages are central to tissue homeostasis (68), resident ATMs’ origin, identity, and kinetics are still not well-understood. This study determined the development of resident macrophage subsets in adipose tissue at a steady state and their response to metabolic challenges. Using extensive marker selection with unsupervised high-dimensional single-cell analyses, we distinguished six different resident ATM populations in lean mice. Four of them expressed CD206, a classical marker for alternatively activated M2-like macrophages, while the two CD206-negative populations were identified by CD11c and Ly6C positivity. In addition, TIM4 and CD163 were identified as dichotomic markers for separating the four major CD206 positive macrophage populations. All four CD206 positive ATM populations were further subdivided by the MHC II expression status and displayed characteristic kinetic changes during aging. Combining cell-fate mapping and macrophage deficient mouse models, we show that resident ATM macrophages represent in lean mice a mixture of cells originating from the yolk sac, fetal liver, and adult bone marrow. Our diet interventions showed that the numbers of CD206 positive resident ATMs in WAT are largely diet independent, yet obesity induced reversible changes in MHC II expression of resident ATMs.

Adipose tissue forms already *in utero*, and new adipocytes are generated continuously at a substantial level, even in adults (69). ATMs are critical during the early postnatal life when *de novo* generation of adipocytes is elevated (12, 69). We found that all major ATM populations were identifiable already at two weeks of age and that the overall number of ATMs grew during postnatal development. While the frequency of the TIM4^+^CD163^+^ and TIM4^+^ subsets decreased upon aging, the two other CD206^+^ macrophage populations, CD163^+^, and TIM4^−^CD163^−^ cells, steadily increased in postnatal mice. Whereas the MHC II negative subpopulations dominated the TIM4^+^CD163^+^ and TIM4^+^ cells at the early postnatal age, MHC II positive subpopulations became dominant in the adults. In contrast, CD163^+^ and TIM4^−^CD163^−^ ATMs were mainly MHC II^+^ throughout postnatal life.

Recently many groups have studied the ATMs in lean mice with different techniques (3, 11, 15, 38–41, 48, 70). While these studies have identified various resident ATM populations in eWAT, comparing the differences between macrophage populations from the different studies has been challenging. Thus, there was an obvious need for a more uniform and precise definition of ATMs before studying the subset-specific function of these heterogenic resident ATMs in the future. Most ATM studies have been done using conventional fluorimetric flow cytometry with a limited set of classical macrophage markers F4/80, CD11b, and CD64 (8, 39, 48), while other studies have used CD11c, CD206, or CX3CR1 instead (3, 15, 41, 71). Major limitations in all of these studies have been the suboptimal marker repertoire for dissecting the true heterogeneity of resident ATMs, challenges in controlling the high autofluorescence of myeloid cells, and the bias introduced by manual gating strategies. More comprehensive but still limited marker selection was used when the vascular-associated macrophage (VAM) nomenclature was proposed (11).

Few RNA studies have also addressed the ATMs in lean mice (38, 40, 70). In scRNAseq analyses of lean mice, Jaitin et al. identified only one macrophage cluster that expressed *Cd163* and *Lyve-1*, markers commonly associated with perivascular macrophages (38, 51). Bulk RNA results of VAM2 confirmed the transcription of the same markers in lean mice (11). In contrast, Weinstock et al. identified six different ATM populations in lean adult mice (40). Two prominent macrophage clusters in lean mice, designed as resident macs and activated macs, prospectively resemble our TIM4^+^CD163^+^ (both MHC II^+^ and MHC II^−^ subpopulations) cells, as *Cd163*, *Lyve-1*, and *H2-Ab* were expressed in these clusters. Interestingly, *Tim4* was not present in differentially expressed genes in any of the clusters (40). One of the clusters identified, named stem-like macs, highly expressed genes related to proliferation, such as *Ube2c*, *Top2a*, and *Mki67*. Likewise, we saw that proportion of TIM4^+^CD163^+^ and CD11c^+^ ATM cells consistently proliferated at both 5 and 16 week time points. From our data, we identified a smaller CD206^−^Ly6C^+^ macrophage population that was TIM3 and CD43 positive. Similar cells have been previously called B cell-like and Heme macs. Moreover, the monocyte cluster identified in scRNAseq analyses highly expressed *Fcgr1* (CD64) and thus likely includes cells similar to our CD206^−^Ly6C^+^ macrophages. In obesity, resident macrophages called major macs were found to expand along with newly appearing phagocytic macs (40). Together, these two clusters likely correspond to our TIM4^−^CD163^−^ ATM population mingling with the incoming CD11c population in obese mice. Weinstock et al. found that only the number of major macs increased in obese animals, while the other resident macrophage clusters remained relatively stable in obese mice, which is consistent with our observations. Clearly, further studies are warranted to precisely correlate RNA and protein levels in ATM macrophages for an unambiguous definition of different subpopulations in lean and obese mice.

Macrophages internalize extracellular antigens through endocytosis and phagocytosis and process them for antigen presentation. Our *in vitro* analyses showed that each of the four main ATM macrophage subtypes is capable of endocytosing and phagocyting several different cargos and are hence bona fide macrophages. *In vitro*, TIM4^+^CD163^+^ and TIM4^+^ macrophages, compared to the other main ATMs, showed an approximately two-fold increase in endocytosis of dextran. However, the *in vivo* binding/endocytosis assays with dextran and immunocomplex revealed TIM4^+^CD163^+^ macrophages as the main interacting population. The result may reflect superior scavenging capacity or physiological exposure of TIM4^+^CD163^+^ cells to the blood-borne ligands. Nevertheless, all macrophage populations contained LYVE1^+^ subsets, tentatively identified as vessel-associated macrophages (72, 73), and all populations had cells highly expressing CD206, which is the primary scavenger receptor for dextran (74, 75). Therefore, other mechanisms may contribute to the better capacity of TIM4^+^CD163^+^ macrophages for *in vivo* scavenging. Of note, *in vitro* endocytosis/phagocytosis assays revealed that also MHC II negative subsets are effective in these processes. It may imply a functional specialization in which the ingested antigens are not presented to boost the immune system but are possibly silently disposed.

Recent studies of macrophage ontogeny have highlighted macrophage heterogeneity and have revealed that various tissue-resident macrophage compartments are already established during embryogenesis (43, 46, 51, 76). Under homeostatic conditions, each adult tissue has its unique composition of coexisting embryonic-derived and adult bone marrow-generated macrophages (55–57, 59, 77). Thus, ATM expansion can be driven by *in situ* proliferation and/or by the influx of new bone marrow-derived monocytic precursors. Our *Ccr2* knockout data collectively suggest that the recruitment of CCR2-mediated monocytes from the blood is crucial for expanding the resident CD163^+^ and TIM4^−^CD163^−^ populations. Our results are well in line with lineage tracking studies done with Ms4a3-Cre-dtTomato reporter, labeling all bone marrow-derived monocytes but not embryonically derived macrophages, showing that 50% of ATMs are bone marrow origin (38). Resident ATMs are proposed to be predominantly derived from embryonic yolk sac precursors (12). Using fate mapping models and *Plvap* deficient mice, we showed that long-lived embryonic-derived, both yolk sac and liver origin, macrophages exist in ATMs but almost solely within the TIM4^+^CD163^+^ subpopulation. These results are consistent with a previous study showing that yolk sac-derived macrophages are necessary to expand adipocytes postnatally (12).

The adaptation of adipose tissue to diet variations is critical to the maintenance of metabolic control. In obese adipose tissue, the high influx of recruited monocyte-derived CD11c^+^ ATMs leads to low-grade systemic chronic inflammation and insulin resistance (19, 66). In line with published data, we observed the HFD induced increase of total macrophages as a cause of the massive expansion of the inflammatory CD11c^+^ ATM cells. However, the cell numbers of resident TIM4^+^CD163^+^, TIM4^+^, and CD163^+^ populations remained surprisingly steady despite the diet or treatment interventions, suggesting a homeostatic role for tissue-resident macrophages in white adipose tissue.

Diet-induced obesity promotes the differentiation of ATMs towards a more classically activated M1-like phenotype reflected by an increased MHC II expression. In contrast to previously published papers (48, 78), our studies revealed that although diet-induced obesity might increase MHC II expression in ATMs generally (3), both TIM4^+^ resident ATM populations decreased their MHC II expression significantly during the HFD. After this phenotypic switch, these cells started to resemble the early postnatal CD206^+^TIM4^+^CD163^+^ MHCII^−^ cells. The loss of MHC II from TIM4^+^ ATMs during HFD notably did not impact antigen processing capability. Remarkably, after eight weeks of the diet switch from HFD to Chow, the TIM4^+^CD163^+^ ATMs had regained the high MHC II expression. Although the functional implications of the phenotypic switch remain to be studied, it may be related to adipose tissue remodeling, to which yolk sac-derived macrophages contribute in early life (79, 80).

In conclusion, we show here that tissue‐resident macrophage subsets in white adipose tissue consist of four main populations defined by CD206, TIM4, and CD163 markers. The contribution of embryonic and adult bone marrow varies in the generation of the distinct macrophage populations, and at least the yolk sac-derived macrophages do not show any tendency towards phenotypic conversion to other macrophage types. Furthermore, tissue-resident macrophage subpopulations respond differently to metabolic challenges. These results imply that both ontogeny-derived and tissue-derived signals control the plasticity of resident ATMs. Moreover, the diet intervention studies suggest that the pro-inflammatory macrophage phenotype induced by HFD is not a fixed state but can be reverted to a normal anti-inflammatory phenotype by dietary therapy. We thus reveal previously unknown heterogeneity of tissue‐resident macrophage subsets in white adipose tissue with advanced single-cell proteomics technology and unbiased single-cell analyses. Our data should be useful for defining individual subsets of resident macrophages when dissecting their metabolic and other functional roles in the future.

## Data Availability Statement

The raw data supporting the conclusions of this article will be made available by the authors, without undue reservation.

## Ethics Statement

The animal study was reviewed and approved by Regional Animal Experiment Board in Finland.

## Author Contributions

IF contributed to experiment design, conducted experiments, analyzed data, and contributed to the preparation of the manuscript. HJ designed experiments, conducted experiments, supervised the study and wrote the manuscript. JK performed experiments and contributed to data analyses. NK and ES provided samples and contributed to the preparation of the manuscript. MS and PR conceived and supervised the study and wrote the manuscript. All authors contributed to the article and approved the submitted version.

## Funding

This study was financially supported by the Academy of Finland, Sigrid Juselius Foundation, Jane and Aatos Erkko Foundation, The Cancer Foundation Finland, and Turku Doctoral Program of Molecular Medicine.

## Conflict of Interest

The authors declare that the research was conducted in the absence of any commercial or financial relationships that could be construed as a potential conflict of interest.

## Publisher’s Note

All claims expressed in this article are solely those of the authors and do not necessarily represent those of their affiliated organizations, or those of the publisher, the editors and the reviewers. Any product that may be evaluated in this article, or claim that may be made by its manufacturer, is not guaranteed or endorsed by the publisher.
