# Supplementary material for: Single-Cell Proteomics Reveals the Defined Heterogeneity of Resident Macrophages in White Adipose Tissue
Source: Front Immunol. 2021 Jul 26;12:719979. doi: 10.3389/fimmu.2021.719979 (PMC8350344; doi:10.3389/fimmu.2021.719979)
Supplement: Supplementary file 1 [file Presentation_1.pdf]

Supplementary Material for

Single-cell proteomics reveals the defined heterogeneity of resident macrophages in white adipose tissue, Félix *et al*.

The Supplementary Material for this article includes 10 Supplementary Figures and 1 Supplementary Table.

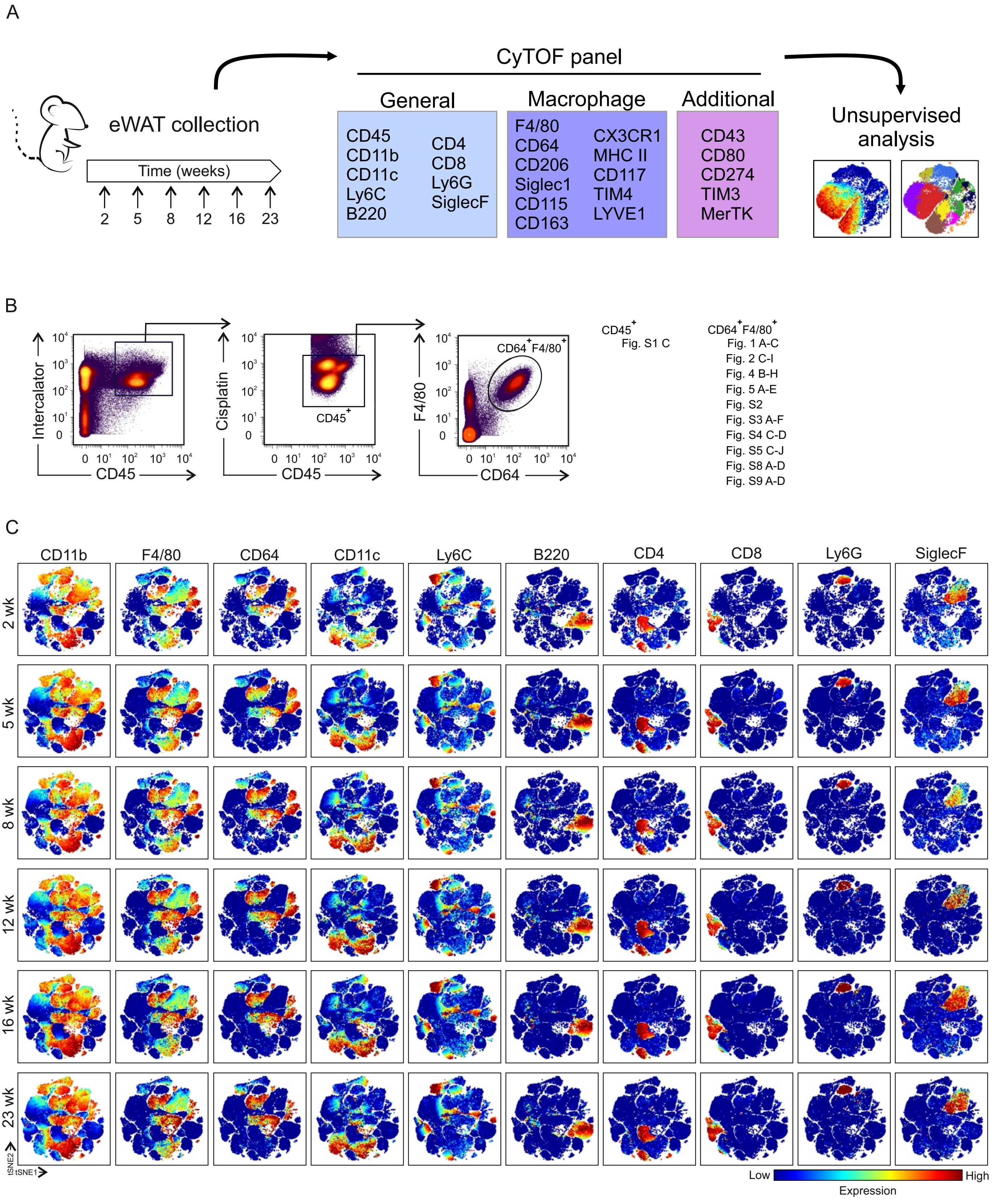
A
CyTOF panel
eWAT collection
Time (weeks)
2 5 8 12 16 23
General
CD45 CD11b CD11c Ly6C B220
CD4 CD8 Ly6G SiglecF
Macrophage
F4/80 CD64 CD206 Siglec1 CD115 CD163
CX3CR1 MHC II CD117 TIM4 LYVE1
Additional
CD43 CD80 CD274 TIM3 MerTK
Unsupervised analysis
B
Intercalator
CD45
Cisplatin
CD45+
F4/80
CD64
CD64+F4/80+
CD45+
Fig. S1 C
CD64+F4/80+
Fig. 1 A-C
Fig. 2 C-I
Fig. 4 B-H
Fig. 5 A-E
Fig. S2
Fig. S3 A-F
Fig. S4 C-D
Fig. S5 C-J
Fig. S8 A-D
Fig. S9 A-D
C
CD11b
F4/80
CD64
CD11c
Ly6C
B220
CD4
CD8
Ly6G
SiglecF
2 wk
5 wk
8 wk
12 wk
16 wk
23 wk
tSNE2
tSNE1
Low
High
Expression

Supplementary Figure 1. (A) Schematic diagram for the experimental design and workflow. **(B)** Representative gating strategy of the mass cytometric data for eWAT single (Intercalation^{+}) live (Cisplatin^{-}) CD45^{+} leukocytes and CD45^{+}CD64^{+}F4/80^{+} macrophages. **(C)** Representative viSNE plots of CD45^{+} leukocytes at the indicated time points from wild type (WT) eWAT. Color code indicates the expression level of a given marker from low (blue) to high (red).

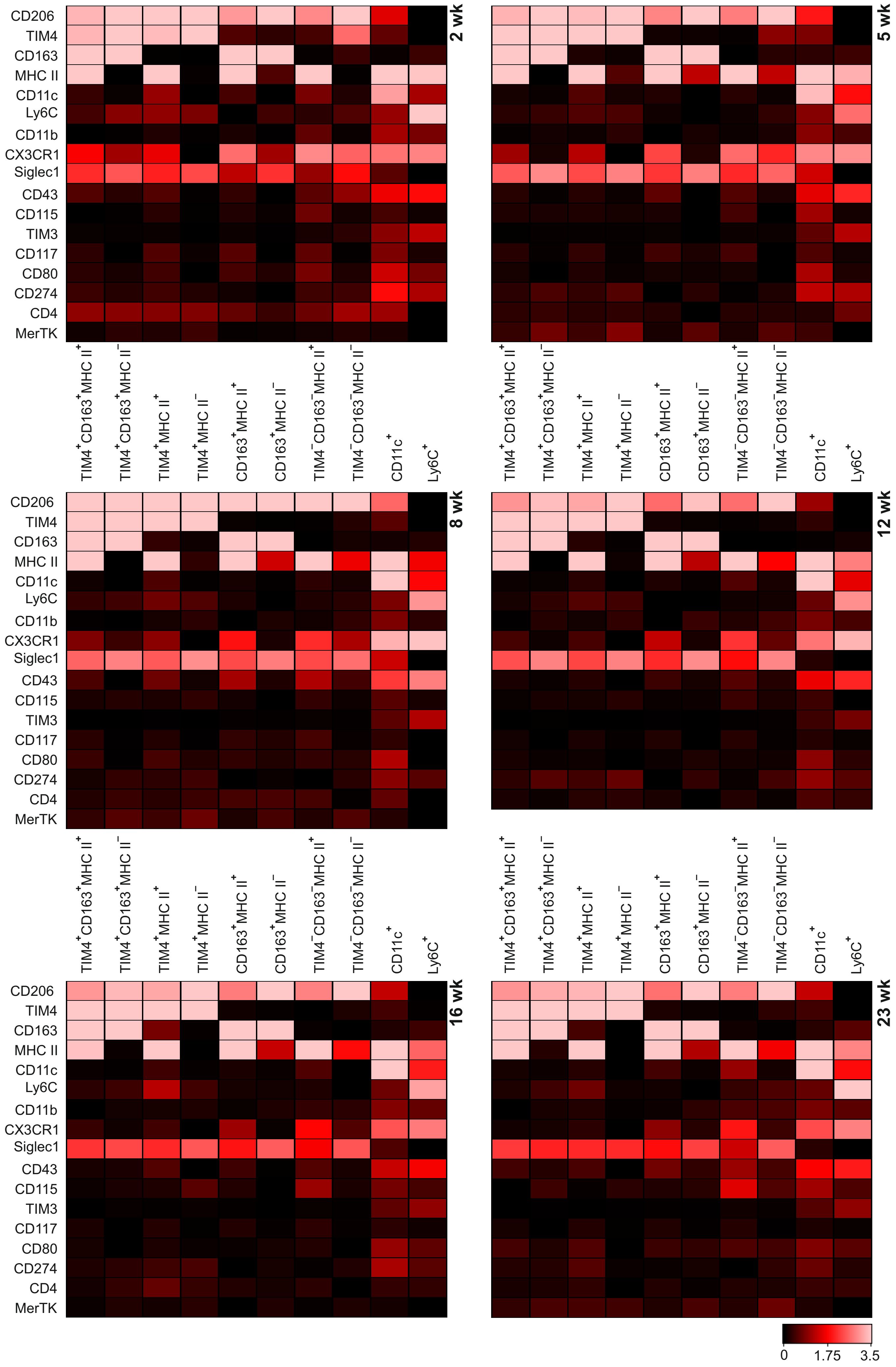
2 wk
5 wk
8 wk
12 wk
16 wk
23 wk
CD206
TIM4
CD163
MHC II
CD11c
Ly6C
CD11b
CX3CR1
Siglec1
CD43
CD115
TIM3
CD117
CD80
CD274
CD4
MerTK
TIM4+CD163+MHC II+
TIM4+CD163+MHC II−
TIM4+MHC II+
TIM4+MHC II−
CD163+MHC II+
CD163+MHC II−
TIM4−CD163−MHC II+
TIM4−CD163−MHC II−
CD11c+
Ly6C+
0
1.75
3.5

Supplementary Figure 2. Representative heat map analyses showing the mean expression of the indicated markers in FlowSOM metaclusters (identified in Figure 1B) in wild type (WT) eWAT at 2, 5, 8, 12, 16, and 23 weeks of age. Color code indicates the expression level of a given marker from low (black) to high (white).

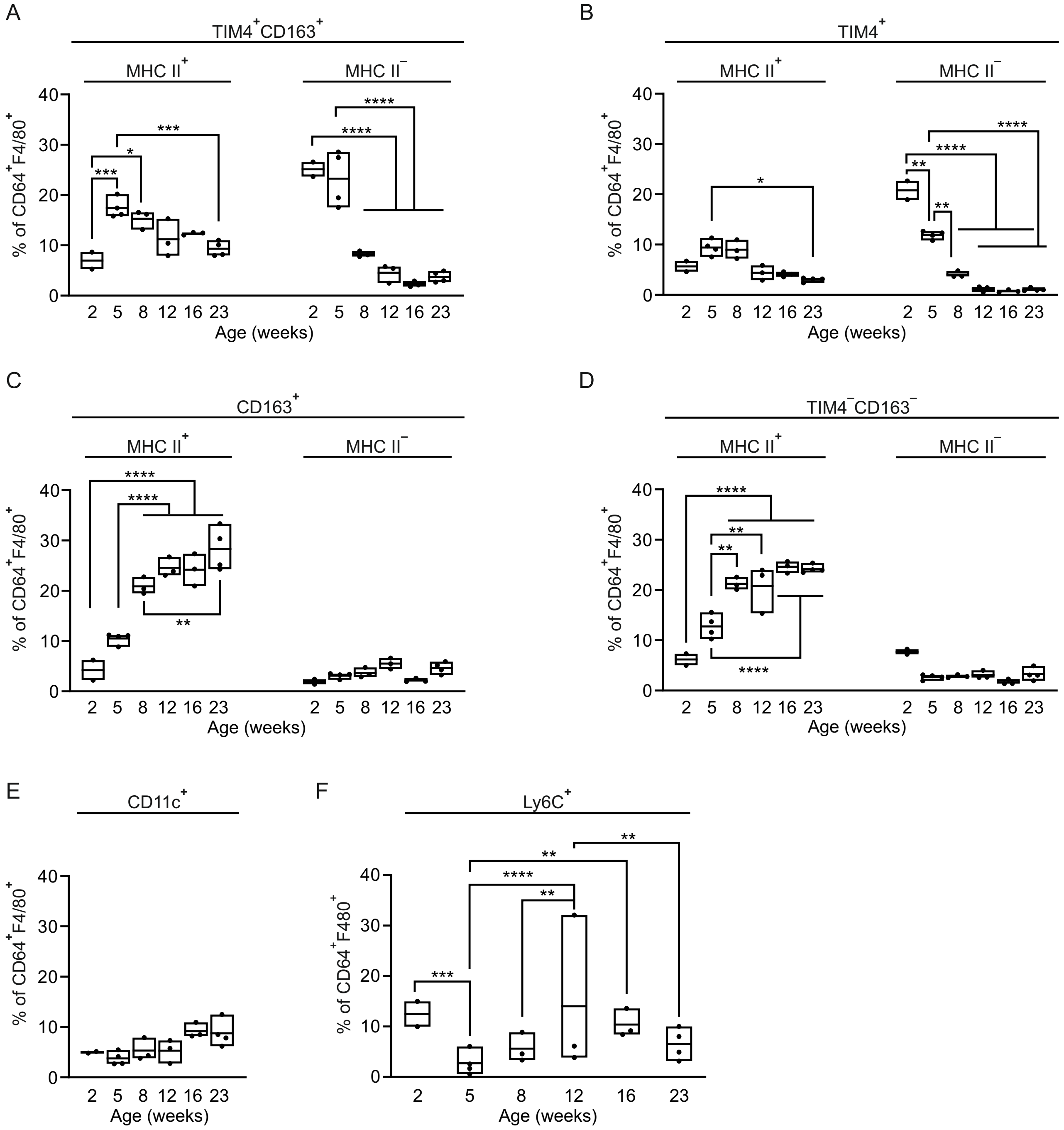

A
TIM4+CD163+
MHC II+
MHC II−
% of CD64+F4/80+
Age (weeks)
B
TIM4+
C
CD163+
D
TIM4−CD163−
E
CD11c+
F
Ly6C+
% of CD64+F480+

Supplementary Figure 3. (A-F) Frequencies of eWAT macrophage subpopulations in wild type (WT) mice based on the FlowSOM analyses at the indicated time points. The quantitative data are shown as mean ± SEM (**P* ≤ 0.0332, ***P* ≤ 0.0021, ****P* ≤ 0.0002, *****P* ≤ 0.0001, two-way ANOVA with Bonferroni post-hoc test). Each data point represents a pooled eWAT from 7 (2 wk), 3 (5 wk), or 2 (8, 12, 16, and 23 wk) mice. All mass cytometry data are from 1 (2, 12 and 16 wk), 2 (8 wk,), 3 (5 wk) or 4 (23 wk) independent experiments.

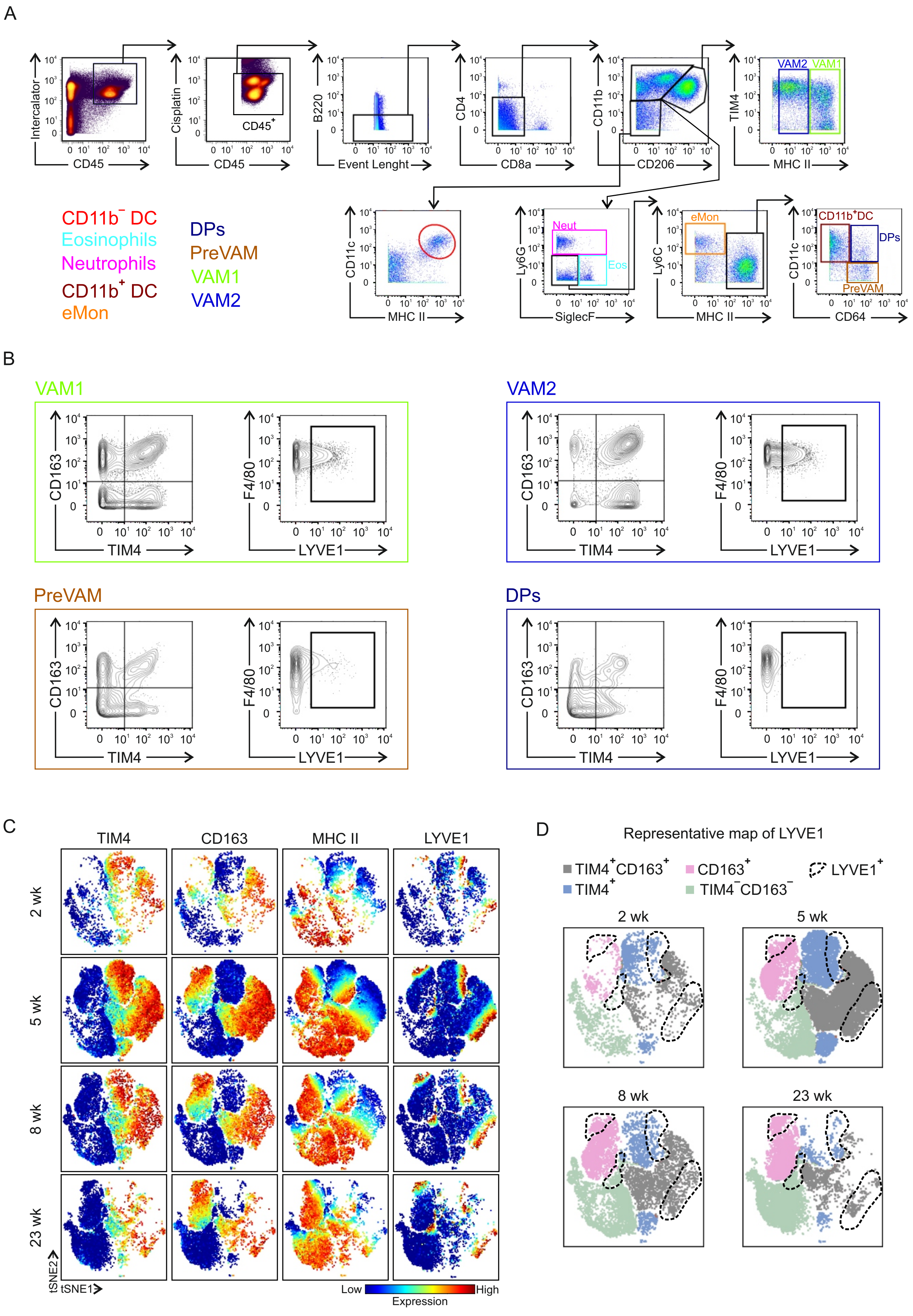

A
Intercalator
CD45
Cisplatin
CD45+
B220
Event Lenght
CD4
CD8a
CD11b
CD206
TIM4
MHC II
VAM2
VAM1
CD11b⁻ DC
Eosinophils
Neutrophils
CD11b⁺ DC
eMon
DPs
PreVAM
VAM1
VAM2
CD11c
MHC II
Ly6G
SiglecF
Neut
Eos
Ly6C
MHC II
eMon
CD11c
CD64
CD11b⁺DC
DPs
PreVAM
B
VAM1
VAM2
PreVAM
DPs
CD163
TIM4
F4/80
LYVE1
C
TIM4
CD163
MHC II
LYVE1
2 wk
5 wk
8 wk
23 wk
tSNE2
tSNE1
Low
High
Expression
D
Representative map of LYVE1
TIM4⁺CD163⁺
CD163⁺
LYVE1⁺
TIM4⁺
TIM4⁻CD163⁻
2 wk
5 wk
8 wk
23 wk

Supplementary Figure 4. (A) Representative example showing the gating of our mass cytometric data from eWAT of a 5-week-old wild type (WT) mouse using the strategy described by Silva *et al*. (11) for defining the vascular-associated macrophages (VAMs). **(B)** TIM4, CD163 and LYVE1 gatings on the different VAM populations. Note that each VAM subpopulation includes cells from $TIM4^{+}CD163^{+}$, $TIM4^{+}$, $CD163^{+}$, and $TIM4^{-}CD163^{-}$ macrophage subpopulations and both $LYVE1^{+}$ and $LYVE1^{-}$ cells. **(C)** Representative eWAT macrophage viSNE plots from WT at the indicated time points showing LYVE1 expression. **(D)** Manual gating of $TIM4^{+}CD163^{+}$, $TIM4^{+}$, $CD163^{+}$, and $TIM4^{-}CD163^{-}$ ATMs on the viSNE map. The areas within the dotted lines (drawn manually based on the data shown in (C) represent LYVE1 positive cells in each main macrophage population.

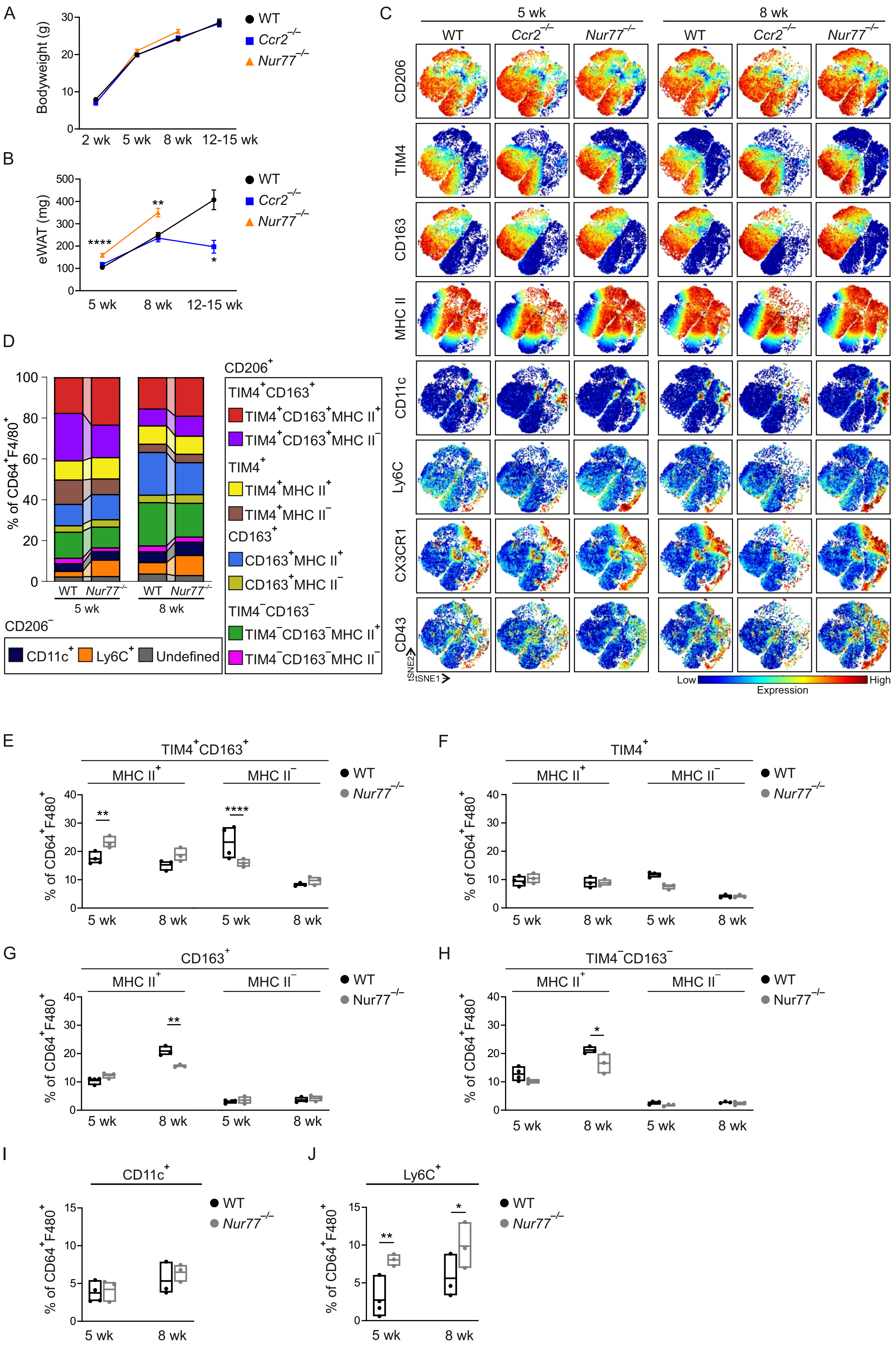

A
Bodyweight (g)
2 wk
5 wk
8 wk
12-15 wk
WT
Ccr2−/−
Nur77−/−
B
eWAT (mg)
****
**
*
C
5 wk
8 wk
WT
Ccr2−/−
Nur77−/−
CD206
TIM4
CD163
MHC II
CD11c
Ly6C
CX3CR1
CD43
tSNE2
tSNE1
Low
High
Expression
D
% of CD64+F4/80+
CD206+
TIM4+CD163+
TIM4+CD163+MHC II+
TIM4+CD163+MHC II−
TIM4+
TIM4+MHC II+
TIM4+MHC II−
CD163+
CD163+MHC II+
CD163+MHC II−
TIM4−CD163−
TIM4−CD163−MHC II+
TIM4−CD163−MHC II−
CD206−
CD11c+
Ly6C+
Undefined
E
TIM4+CD163+
MHC II+
MHC II−
% of CD64+F480+
F
TIM4+
G
CD163+
Nur77−/−
H
TIM4−CD163−
I
CD11c+
J
Ly6C+

Supplementary Figure 5. (A) Bodyweight follow-up of wild type (WT), *Ccr2*$^{-/-}$ and *Nur77*$^{-/-}$mice at the indicated time points. **(B)** eWAT weight of WT, *Ccr2*$^{-/-}$ and *Nur77*$^{-/-}$ mice at indicated time points. **(C)** Representative viSNE plots of eWAT in 5- and 8-week-old WT, *Ccr2*$^{-/-}$ and *Nur77*$^{-/-}$ mice. Color code indicates the expression level of a given marker from low (blue) to high (red). **(D)** Frequencies of macrophage subpopulations in eWAT based on the FlowSOM analyses of *Nur77*$^{-/-}$ mice at 5 and 8 weeks of age. Each FlowSOM metacluster (subpopulation) is represented by an individual color depicted in the columns. **(E-J)** Frequencies of eWAT macrophage subpopulations in *Nur77*$^{-/-}$ mice based on the FlowSOM analyses at the indicated time points The quantitative data are shown as mean ± SEM [*$P \leq 0.0332$, **$P \leq 0.0021$, ***$P \leq 0.0002$, ****$P \leq 0.0001$, non-parametric two-tailed Mann-Whitney test (A and B), or two-way ANOVA with Bonferroni post-hoc test for (E-J)]. Each data point represents a grouped mean value (A and B), a pooled eWAT from 2 (8 wk), or 3 (5 wk) mice (E-J). All mass cytometry data are from 2 (8 wk WT and *Nur77*$^{-/-}$, and 5 wk *Nur77*$^{-/-}$) or 3 (5 wk WT) independent experiments.

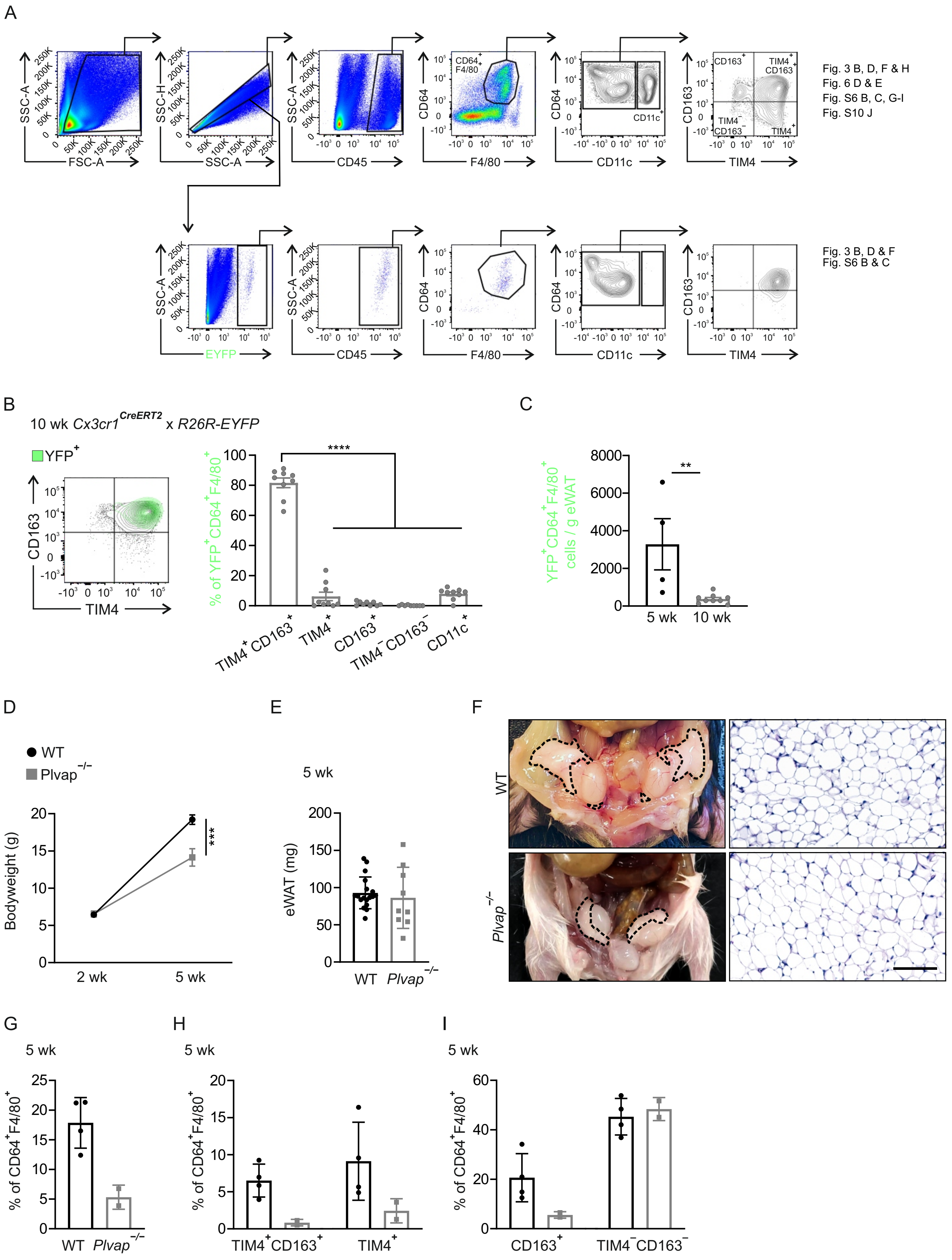

A
SSC-A
FSC-A
SSC-H
SSC-A
CD45
CD64
F4/80
CD64+ F4/80+
CD11c
CD11c+
CD163
TIM4
CD163+
TIM4+ CD163+
TIM4− CD163−
TIM4+
Fig. 3 B, D, F & H
Fig. 6 D & E
Fig. S6 B, C, G-I
Fig. S10 J
EYFP
Fig. 3 B, D & F
Fig. S6 B & C
B
10 wk Cx3cr1CreERT2 x R26R-EYFP
YFP+
% of YFP+CD64+F4/80+
****
TIM4+ CD163+
TIM4+
CD163+
TIM4− CD163−
CD11c+
C
YFP+CD64+F4/80+ cells / g eWAT
**
5 wk
10 wk
D
WT
Plvap−/−
Bodyweight (g)
***
2 wk
5 wk
E
5 wk
eWAT (mg)
WT
Plvap−/−
F
WT
Plvap−/−
G
5 wk
% of CD64+F4/80+
WT
Plvap−/−
H
5 wk
% of CD64+F4/80+
TIM4+CD163+
TIM4+
I
5 wk
% of CD64+F4/80+
CD163+
TIM4−CD163−

Supplementary Figure 6. (A) Representative gating strategy for the flow cytometric data used to identify the four main populations of eWAT macrophages and the strategy for gating YFP positive cells onto the macrophages. **(B)** Representative FACS plot and quantifications of yolk sac-derived cell in eWAT of 10-week-old *Cx3cr1*^{CreERT2}*;R26R-EYFP* reporter mice induced E13.5 with tamoxifen. FACS plot shows the backgating of the YFP$^+$ cells (green) on the different ATM populations. Quantifications show the frequency of CD45$^+$YFP$^+$CD64$^+$F4/80$^+$ cells in each ATM population. **(C)** Quantifications of CD45$^+$YFP$^+$CD64$^+$F4/80$^+$ cell counts at 5 and 10 weeks of age in eWAT of *Cx3cr1*^{CreERT2}*;R26R-EYFP* reporter mice induced E13.5 with tamoxifen. **(D)** Bodyweight (g) of wild type (WT) and *Plvap*$^{-/-}$ male mice at the age of 2 and 5 weeks. **(E)** eWAT weight in 5-week-old WT and *Plvap*$^{-/-}$ mice. **(F)** Representative macroscopic images and PAS-stained histology of eWAT in 5-week-old WT and *Plvap*$^{-/-}$ mice. Epididymal fat pads are outlined with a dashed line. Scale bar 100 µm. **(G)** Quantification of macrophages (CD45$^+$CD64$^+$F4/80$^+$ cells) in eWAT of 5-week-old *Plvap*$^{-/-}$ mice. **(H-I)** Quantification of TIM4$^+$CD163$^+$, TIM4$^+$, CD163$^+$ and TIM4$^-$CD163$^-$ ATMs in eWAT of 5-week-old *Plvap*$^{-/-}$ mice. The quantitative data are shown as mean ± SEM (*$P \leq 0.0332$, **$P \leq 0.0021$, ***$P \leq 0.0002$, ****$P \leq 0.0001$, one-way ANOVA with Bonferroni post-hoc test for B and non-parametric two-tailed Mann-Whitney test for C, D and E). Each data point represents one mouse (B, C, E, G, H and I) or mean value (D). All flow cytometry data are from 2 (G-I for *Plvap*$^{-/-}$) or 3 (B, C, and G-I for WT) independent experiments.

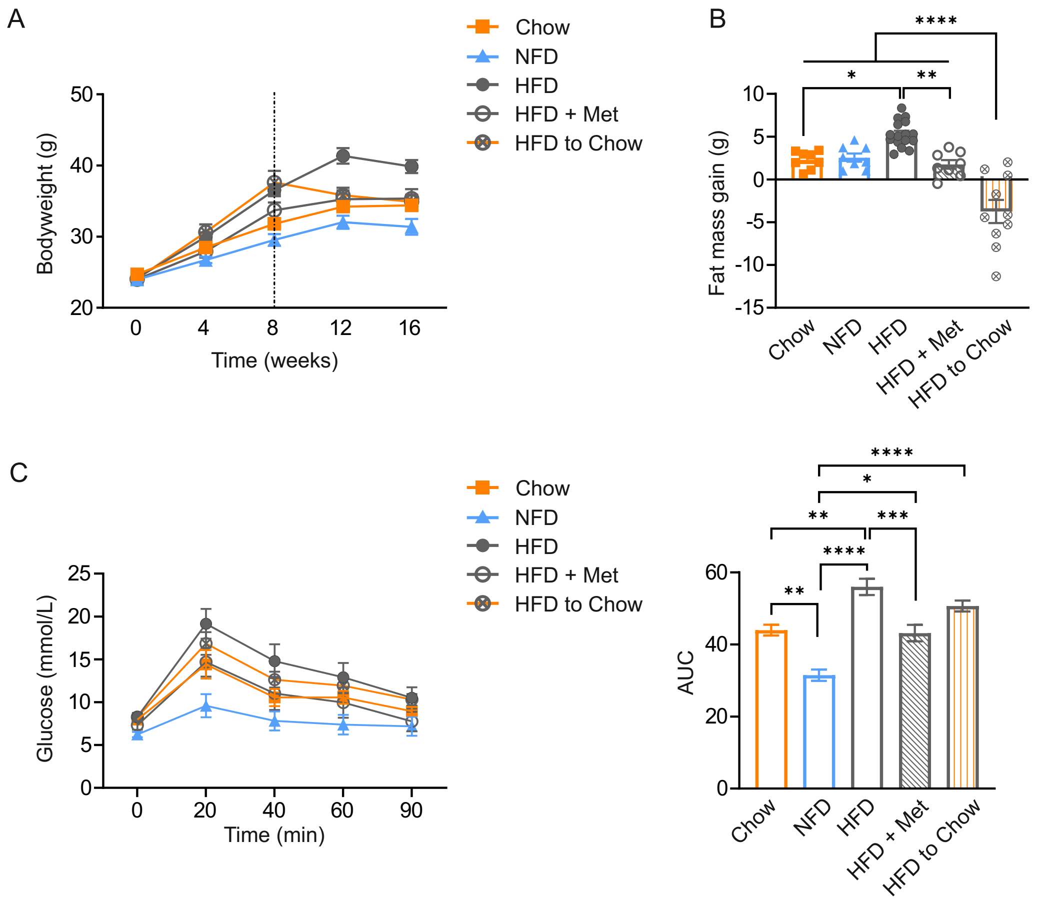
A
Chow
NFD
HFD
HFD + Met
HFD to Chow
Bodyweight (g)
50
40
30
20
0
4
8
12
16
Time (weeks)
B
Fat mass gain (g)
10
5
0
-5
-10
-15
*
**
****
Chow
NFD
HFD
HFD + Met
HFD to Chow
C
Glucose (mmol/L)
25
20
15
10
5
0
0
20
40
60
90
Time (min)
AUC
60
40
20
0
**
****
**
***
*
****
Chow
NFD
HFD
HFD + Met
HFD to Chow

Supplementary Figure 7. (A) Bodyweight gain of wild type (WT) mice in different diet groups. The vertical black line indicates the intervention time in HFD+Met and HFD to Chow groups. **(B)** Quantification of fat mass gain by Echo-MRI between weeks 7 and 15 in the different groups, each dot represents one mouse. **(C)** Glucose tolerance test curves and area under the curve (AUC) values for mice in the different diet groups. The quantitative data are shown as mean ± SEM (*$P \leq 0.0332$, **$P \leq 0.0021$, ***$P \leq 0.0002$, ****$P \leq 0.0001$, one-way ANOVA with Bonferroni post-hoc test).

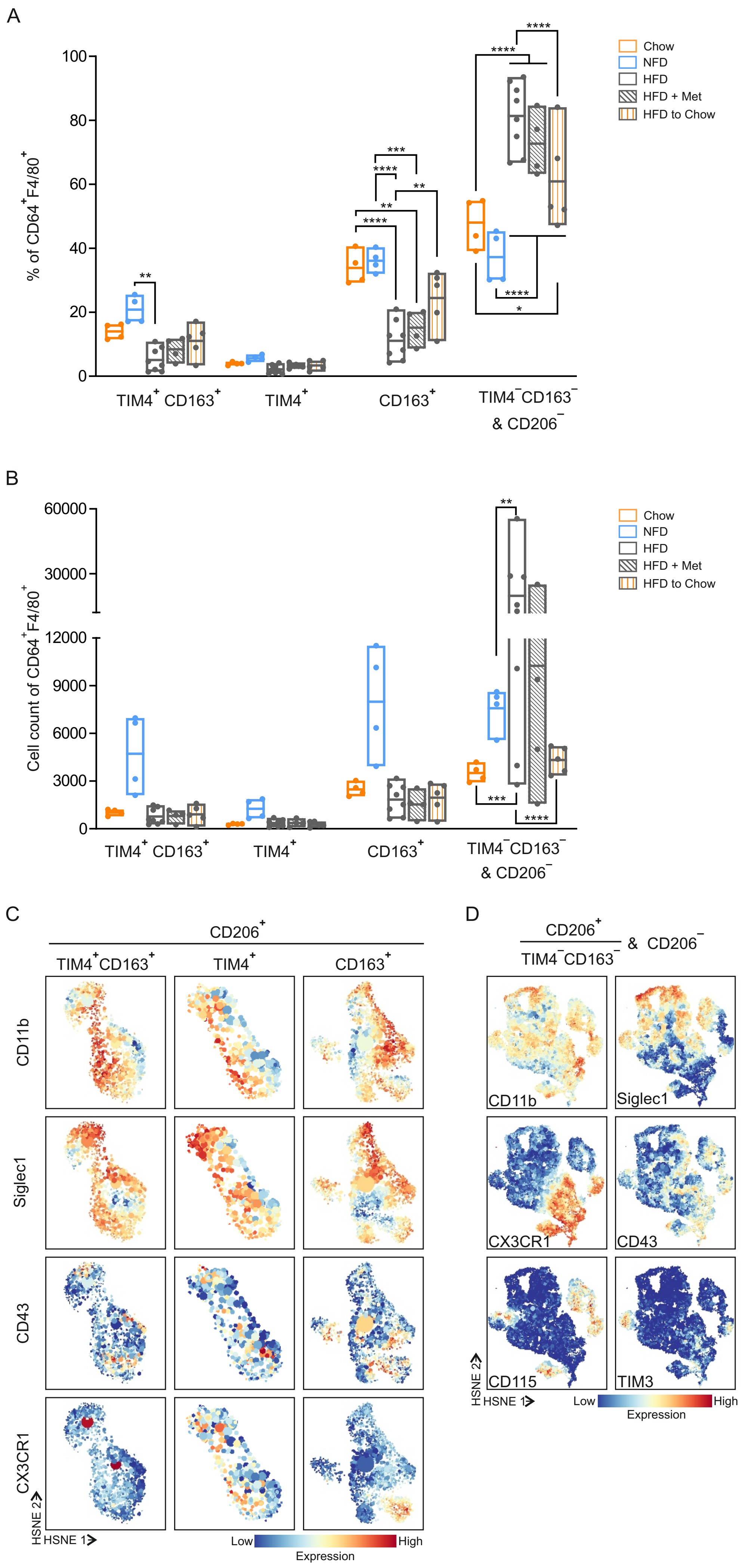
A
Chow
NFD
HFD
HFD + Met
HFD to Chow
% of CD64+F4/80+
TIM4+ CD163+
TIM4+
CD163+
TIM4−CD163−
& CD206−
B
Cell count of CD64+F4/80+
C
CD206+
TIM4+CD163+
TIM4+
CD163+
CD11b
Siglec1
CD43
CX3CR1
HSNE 2
HSNE 1
Low
High
Expression
D
CD206+
TIM4−CD163−
& CD206−
CD11b
Siglec1
CX3CR1
CD43
CD115
TIM3

Supplementary Figure 8. (A) Frequencies of macrophage subpopulations in eWAT of Chow, NFD, HFD, HFD + Met, and HFD to Chow mice at the end of the 16 wk experiment. **(B)** Cell count of macrophage subpopulations in eWAT of Chow, NFD, HFD, HFD + Met, and HFD to Chow mice at the end of the 16 wk experiment. **(C)** Expression level of the indicated marker from low (blue) to high (red) in a selection of $TIM4^{+}CD163^{+}$, $TIM4^{+}$, and $CD163^{+}$ HSNEs. **(D)** Expression level of the indicated markers from low (blue) to high (red) in a selection of $TIM4^{-}CD163^{-}$ and $CD206^{-}$ HSNE. In C and D, the clusters are the same as shown in main Figures 4E and F, respectively. The quantitative data are shown as mean ± SEM (*$P \leq 0.0332$, **$P \leq 0.0021$, ***$P \leq 0.0002$, ****$P \leq 0.0001$, two-way ANOVA with Bonferroni post-hoc test). One data point represents a pooled eWAT from 2 mice. All mass cytometry data are from 3 (HFD + Met and NFD), 4 (Chow and HFD to Chow) or 6 (HFD) independent experiments (A and B).

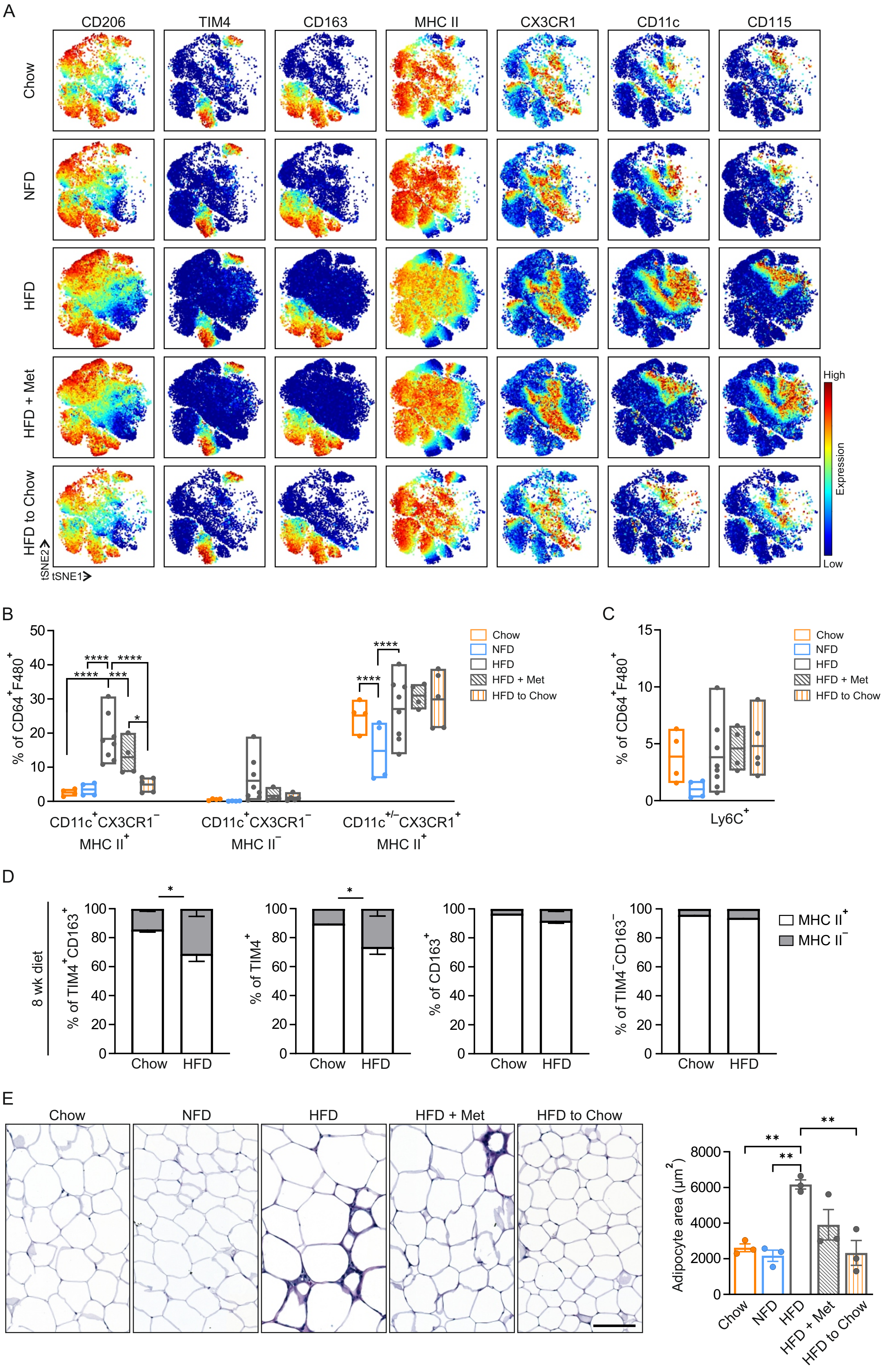
A
CD206
TIM4
CD163
MHC II
CX3CR1
CD11c
CD115
Chow
NFD
HFD
HFD + Met
HFD to Chow
tSNE2
tSNE1
High
Expression
Low
B
% of CD64+F480+
****
***
*
CD11c+CX3CR1− MHC II+
CD11c+CX3CR1− MHC II−
CD11c+/−CX3CR1+ MHC II+
Chow
NFD
HFD
HFD + Met
HFD to Chow
C
% of CD64+F480+
Ly6C+
D
8 wk diet
% of TIM4+CD163+
% of TIM4+
% of CD163+
% of TIM4−CD163−
Chow
HFD
MHC II+
MHC II−
E
Chow
NFD
HFD
HFD + Met
HFD to Chow
Adipocyte area (μm2)
**

Supplementary Figure 9. (A) Representative viSNE plots for indicated diet groups from wild type (WT) eWAT of $CD45^+CD64^+F4/80^+$ cells. Color code indicates the expression level of a given marker from low (blue) to high (red). **(B)** Frequencies of the $CD11c^+CX3CR1^-MHC\ II^+$, $CD11c^+CX3CR1^-MHC\ II^-$ and $CD11c^{+/-}CX3CR1^+MHC\ II^+$ subpopulations in the indicated experimental groups. **(C)** Frequencies of the $Ly6C^+$ cells in the indicated experimental groups. **(D)** Frequencies of $MHC\ II^+$ and $MHC\ II^-$ cells in $TIM4^+CD163^+$, $TIM4^+$, $CD163^+$, and $TIM4^-CD163^-$ ATMs in mice after 8 weeks on Chow or HFD. **(E)** Representative PAS stained eWAT histology and quantification of adipocyte area (μm^2) of the indicated experimental groups. Scale bar 100 µm. The quantitative data are shown as mean ± SEM [$*P \leq 0.0332$, $**P \leq 0.0021$, $***P \leq 0.0002$, $****P \leq 0.0001$, one- (E) or two-way (B-D) ANOVA with Bonferroni post-hoc test]. Each data point represents 1 mouse (E) or a pooled eWAT from 2 (B and C) mice. 8 wk diet experiment (D) data are from n = 5 (HFD) or n = 6 (Chow) mice. All mass cytometry data are from 1 (Chow and HFD 8wk diet), 3 (HFD + Met and NFD), 4 (Chow and HFD to Chow) or 6 (HFD) independent experiments.

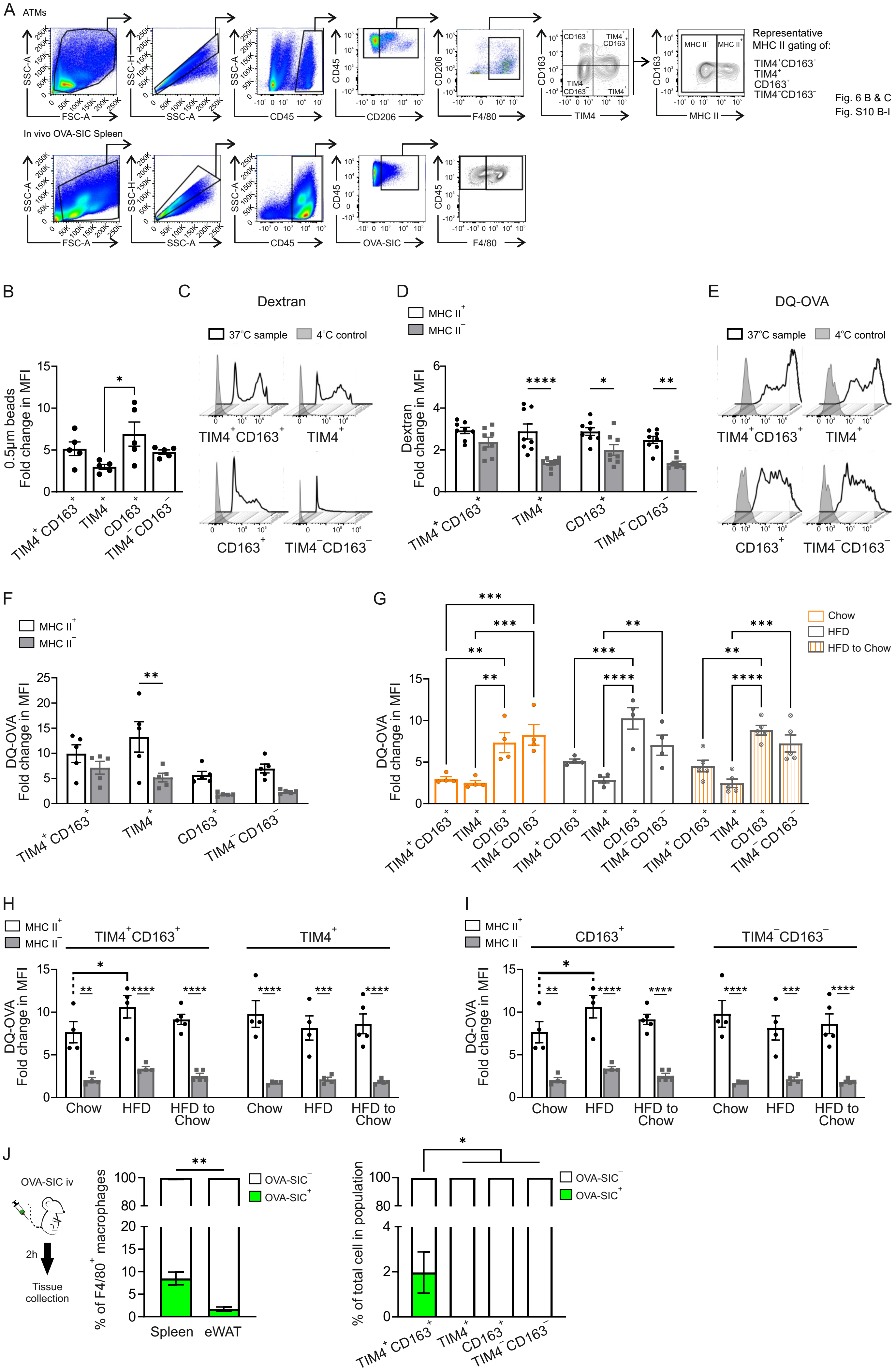
A ATMs
FSC-A
SSC-A
SSC-H
CD45
CD206
F4/80
CD163
TIM4
MHC II
Representative MHC II gating of:
TIM4+CD163+
TIM4+
CD163+
TIM4−CD163−
Fig. 6 B & C
Fig. S10 B-I
In vivo OVA-SIC Spleen
OVA-SIC
B
0.5μm beads Fold change in MFI
C
Dextran
37°C sample
4°C control
D
MHC II+
MHC II−
Dextran Fold change in MFI
E
DQ-OVA
F
DQ-OVA Fold change in MFI
G
Chow
HFD
HFD to Chow
H
I
J
OVA-SIC iv
2h
Tissue collection
% of F4/80+ macrophages
OVA-SIC−
OVA-SIC+
Spleen
eWAT
% of total cell in population

Supplementary Figure 10. (A) Representative gating strategy for the flow cytometric data used for gating cells onto the four main macrophage populations and their MHC II^{+} and MHC II$^{–}$ subtypes, and gating strategy for the spleen CD45^{+}F4/80^{+} cells. **(B)** Phagocytosis of particles by eWAT macrophages. Fold change in mean fluorescence intensity (MFI) in TIM4^{+}CD163^{+}, TIM4^{+}, CD163^{+}, and TIM4$^{–}$CD163$^{–}$ eWAT macrophages after *in vitro* phagocytosis of fluorescent 0.5 µm beads. **(C)** Representative histograms for dextran in four main ATM populations in 5-week-old wild type (WT) mice. **(D)** *In vitro* endocytosis of dextran by MHC II^{+} and MHC II$^{–}$ macrophage subpopulations. Fold change in dextran MFI in MHC II^{+} and MHC II$^{–}$ subsets of TIM4^{+}CD163^{+}, TIM4^{+}, CD163^{+}, and TIM4$^{–}$CD163$^{–}$ eWAT macrophages. **(E)** Representative histograms for DQ-OVA in four main ATM populations in 5-week-old WT mice. **(F)** *In vitro* antigen processing by MHC II^{+} and MHC II$^{–}$ macrophage subpopulations. Fold change in DQ-OVA MFI in MHC II^{+} and MHC II$^{–}$ subsets of TIM4^{+}CD163^{+}, TIM4^{+}, CD163^{+}, and TIM4$^{–}$CD163$^{–}$ eWAT macrophages. **(G)** *In vitro* antigen processing by eWAT macrophages of Chow, HFD, and HFD to Chow mice. **(H-I)** *In vitro* antigen processing by MHC II^{+} and MHC II$^{–}$ macrophage subpopulations. Fold change in DQ-OVA MFI in MHC II^{+} and MHC II$^{–}$ subsets of TIM4^{+}CD163^{+}, TIM4^{+}, CD163^{+}, and TIM4$^{–}$CD163$^{–}$ eWAT macrophages. **(J)** Experimental setup for intravenously administered ovalbumin immune complex (OVA-SIC) uptake and quantifications of OVA-SIC positive and negative macrophage frequencies in the spleen (CD45^{+}F4/80^{+}cells) and eWAT (CD45^{+}CD64^{+}F4/80^{+}cells). Quantifications of OVA-SIC positive and negative cell frequencies in TIM4^{+}CD163^{+}, TIM4^{+}, CD163^{+}, and TIM4$^{–}$CD163$^{–}$ ATM populations of 5-week-old WT mice under *in vivo* settings. The quantitative data are shown as mean ± SEM [*$P \leq 0.0332$, **$P \leq 0.0021$, ***$P \leq 0.0002$, ****$P \leq 0.0001$, one-way ANOVA (B and for ATM population comparison in J) and two-way ANOVA with Bonferroni post-hoc test (D, F, G, H and I), and Mann-Whitney test (for spleen and eWAT comparison in J). In quantifications, data are presented as fold change in MFI (at 37 °C versus background at 4 °C), and each dot represents one mouse. OVA-SIC *in vivo* experiment (J) data are from n = 9 mice. All flow cytometry data are from 2 (B, D, F and J) or 3 (G-I) independent experiments.

Supplementary Table 1 – Antibody list

**Antibody**	**Manufactor**	**Catalog number and RRID ID**
anti-APC 176Yb	Fluidigm	Cat#: 3176007C
anti-B220 160Gd	Fluidigm	Cat#: 3160012C
anti-CD4 172Yb	Fluidigm	Cat#: 3172003C
anti-CD8a 168Er	Fluidigm	Cat#: 3168003C
anti-CD11b APC-Cy-7	BD Pharmingen™	Cat#: BD 557657 RRID: AB_396772
anti-CD11b BV786	BD Pharmingen™	Cat#: BD 740861 RRID: AB_2740514
anti-CD11b 148Nd	Fluidigm	Cat#: 3148003C
anti-CD11c BV711	BioLegend®	Cat#: 117349 RRID: AB_2563905
anti-CD11c 142Nd	Fluidigm	Cat#: 3142003C
anti-CD16/32	BioXCell	Cat#: BE0307 RRID: AB_2736987
anti-CD19 166Er	Fluidigm	Cat#: 3166015C
anti-CD43 146Nd	Fluidigm	Cat#: 3146009C
anti-CD45 PerCP-Cy5,5	BD Pharmingen™	Cat#:BD 550994 RRID: AB_394003
anti-CD45 147Sm	Fluidigm	Cat#: 3147003C
anti-CD45 175Lu	Fluidigm	Cat#: 3175010C
anti-CD45 89Y	Fluidigm	Cat#: 3089005C
anti-CD64 BV786	BD Pharmingen™	Cat#: BD 741024 RRID: AB_2740644
anti-CD64 PE	BioLegend®	Cat#: 139304 RRID: AB_10612740
anti-CD64 151Eu	Fluidigm	Cat#: 3151012C
anti-CD68 FITC	BioLegend®	Cat#: 137005 RRID: AB_10575475
anti-CD80 171Yb	Fluidigm	Cat#: 3171008C
anti-CD115	Bio X Cell	Cat#: BE0213 RRID: AB_2687699
anti-CD115 PE-Cy7	eBioscience	Cat#: 25-1152-80 RRID: AB_2573385
anti-CD115 144Nd	Fluidigm	Cat#: 3144012C
anti-CD117 173Yb	Fluidigm	Cat#: 3173004C
anti-CD163	BioRad	Cat#: MCA342GA RRID: AB_2074558
anti-CD163 APC	BioLegend®	Cat#: 155305 RRID: AB_2814059
anti-CD206 A488	BioRad	Cat#: MCA2235A488T RRID: AB_2297790
anti-CD206 BV650	BioLegend®	Cat#: 141723RRID: AB_2562445
anti-CD206 169Tm	Fluidigm	Cat#: 3169021C
anti-CD274 153Eu	Fluidigm	Cat#: 3153016C
anti-Chicken Egg Albumin	Sigma-Aldrich	Cat#: C6534-2ML
anti-Cisplatin 195Pt	Fluidigm	Cat#: 201064
anti-CX3CR1 BV650	BioLegend®	Cat#: 149033 RRID: AB_2565999
anti-CX3CR1 164Dy	Fluidigm	Cat#: 3164023C
anti-F4/80 A488	eBioscience	Cat#: 53-4801-82 RRID: AB_469915
anti-F4/80 A700	BioRad	Cat#: MCA497A700T RRID:AB_1102556
anti-F4/80 159Tb	Fluidigm	Cat#: 3159009C
anti-FITC 144Nd	Fluidigm	Cat#: 3144006C
anti-Intercalator 103Rh	Fluidigm	Cat#: 201103A
anti-Ly6C BV421	BD Pharmingen™	Cat#: BD 562727 RRID: AB_2737748
anti-Ly6C 150Nd	Fluidigm	Cat#: 3150010C
anti-Ly6C 162Dy	Fluidigm	Cat#: 3162014C
anti-Ly6G BV510	BioLegend®	Cat#: 127633 RRID: AB_2562937
anti-Ly6G 141Pr	Fluidigm	Cat#: 3141008C
anti-LYVE1 PE	R&D Systems	Cat#: FAB2125P RRID: AB_10889020
anti-MerTK APC	R&D Systems	Cat#: FAB5912A

Supplementary Table 1 – Antibody list

Antibody	Manufactor	Catalog number and RRID ID
anti-MerTK FITC	BioLegend®	Cat#: 151503 RRID: AB_2617034
anti-MHC II APC	eBioscience	Cat#: 17-5321-82 RRID: AB_469455
anti-MHC II PE-Cy7	eBioscience	Cat#: 25-5321-82 RRID: AB_10870792
anti-MHC II 174Yb	Fluidigm	Cat#: 3174003C
anti-PE 165Ho	Fluidigm	Cat#: 3165015C
anti-SiglecF APC	BioLegend®	Cat#: 155507 RRID: AB_2750236
anti-SiglecF PE-CF594	BD Pharmingen™	Cat#: BD 562757 RRID: AB_2687994
anti-Siglec1 170Er	Fluidigm	Cat#: 3170018C
anti-TER-119 154Sm	Fluidigm	Cat#: 3154005C
anti-TIM3 162Dy	Fluidigm	Cat#: 3162029C
anti-TIM4	BioLegend®	Cat#: 130002 RRID: AB_1227802
anti-TIM4 PE	BioLegend®	Cat#: 130005 RRID: AB_1227807
